# Supplementary material for: Health and well-being of male international migrants and non-migrants in Bangladesh: A cross-sectional follow-up study
Source: PLoS Med. 2020 Mar 31;17(3):e1003081. doi: 10.1371/journal.pmed.1003081 (PMC7108692; doi:10.1371/journal.pmed.1003081)
Supplement: S1 Table — (DOCX) [file pmed.1003081.s002.docx]

**S1 Table. Full model specifications for all dependent variables.**

Controls for model specifications

Model 1 – Migration status + Age

Model 2 – Adding individual controls

Model 3 – Adding family controls

Model 4 – Adding father/sibling migration controls

Model 5 – Adding controls from 1982 census

| Full models, OLS regression of annual income | | | | | |
| --- | --- | --- | --- | --- | --- |
| VARIABLES | (1) | (2) | (3) | (4) | (5) |
|  |  |  |  |  |  |
| m2_mig_status2 = 0, Non-migrant | -3,951.9 | -3,827.8 | -3,810.1 | -3,797.9 | -3,742.4 |
|  | (216.4)*** | (211.8)*** | (213.7)*** | (213.2)*** | (231.3)*** |
| m2_mig_status2 = 1, Internal migrant | -3,220.7 | -3,270.9 | -3,239.8 | -3,219.3 | -3,090.2 |
|  | (218.2)*** | (220.3)*** | (222.5)*** | (230.0)*** | (243.9)*** |
| m2_mig_status2 = 3, Return int'l migrant | -3,474.5 | -3,509.7 | -3,528.0 | -3,532.3 | -3,566.6 |
|  | (296.8)*** | (282.8)*** | (285.6)*** | (285.8)*** | (305.4)*** |
| Age 25-29 | 675.5 | 717.2 | 675.2 | 668.9 | 641.5 |
|  | (159.9)*** | (154.8)*** | (150.6)*** | (154.6)*** | (147.7)*** |
| Age 30-34 | 938.4 | 909.7 | 819.8 | 802.2 | 848.9 |
|  | (154.8)*** | (166.2)*** | (175.6)*** | (174.9)*** | (198.2)*** |
| Age 35-39 | 802.7 | 710.6 | 549.7 | 525.1 | 544.7 |
|  | (136.5)*** | (146.1)*** | (160.9)*** | (162.6)** | (177.4)** |
| Age 40-44 | 1,027.8 | 1,103.4 | 928.3 | 899.9 | 939.5 |
|  | (302.1)*** | (310.9)*** | (268.7)*** | (260.0)*** | (244.8)*** |
| Age 45-49 | 709.5 | 920.8 | 708.9 | 680.6 | 509.9 |
|  | (202.9)*** | (223.1)*** | (241.5)** | (243.9)** | (245.2)* |
| Age 50+ | 495.5 | 709.7 | 516.4 | 492.7 | 480.3 |
|  | (175.4)** | (197.2)*** | (204.6)* | (204.2)* | (226.2)* |
| Hindu religion |  | -14.9 | 20.9 | 24.4 | -75.1 |
|  |  | (123.7) | (121.0) | (119.4) | (166.6) |
| Schooling - 1-4 years |  | 159.2 | 104.2 | 100.8 | 140.0 |
|  |  | (97.2) | (100.4) | (100.5) | (92.9) |
| Schooling - 5-9 years |  | 433.3 | 304.0 | 298.7 | 369.7 |
|  |  | (127.0)*** | (134.4)* | (132.3)* | (138.1)** |
| Schooling - Lower Secondary |  | 903.9 | 711.8 | 707.3 | 804.7 |
|  |  | (345.2)** | (341.0)* | (339.0)* | (369.1)* |
| Schooling - Upper Secondarry |  | 890.9 | 686.7 | 675.9 | 635.4 |
|  |  | (246.1)*** | (293.0)* | (296.3)* | (304.7)* |
| Schooling - College |  | 1,784.8 | 1,602.7 | 1,598.2 | 1,237.5 |
|  |  | (331.7)*** | (337.8)*** | (340.3)*** | (257.4)*** |
| Height (cm) |  | 18.9 | 16.9 | 17.1 | 20.9 |
|  |  | (9.3)* | (9.6)+ | (9.6)+ | (9.9)* |
| Household asssets, 1996 (logged) |  |  | 95.1 | 90.6 | 52.7 |
|  |  |  | (38.4)* | (37.6)* | (44.8) |
| Mother's schooling = 1, 1-4 years |  |  | -50.8 | -51.9 | -178.0 |
|  |  |  | (189.1) | (189.4) | (174.4) |
| Mother's schooling = 2, 5-9 years |  |  | -230.1 | -226.6 | -190.7 |
|  |  |  | (230.1) | (233.3) | (247.8) |
| Mother's schooling = 3, 10+ years |  |  | -1,191.5 | -1,179.9 | -1,495.6 |
|  |  |  | (442.4)** | (446.3)** | (467.9)** |
| Father's schooling = 1, 1-4 years |  |  | 96.3 | 104.8 | 152.9 |
|  |  |  | (146.4) | (149.9) | (163.1) |
| Father's schooling = 2, 5-9 years |  |  | 340.0 | 342.7 | 283.5 |
|  |  |  | (180.8)+ | (180.7)+ | (169.8)+ |
| Father's schooling = 3, 10+ years |  |  | 268.3 | 272.9 | 206.6 |
|  |  |  | (247.4) | (248.2) | (297.2) |
| Younger brothers |  |  | 87.1 | 75.6 | 63.0 |
|  |  |  | (63.0) | (62.5) | (63.7) |
| Older brothers |  |  | 86.1 | 70.9 | -22.4 |
|  |  |  | (94.6) | (104.9) | (58.3) |
| Younger sisters |  |  | 23.8 | 24.9 | 8.6 |
|  |  |  | (43.7) | (43.4) | (45.3) |
| Older sisters |  |  | -76.2 | -79.3 | -97.1 |
|  |  |  | (56.9) | (57.6) | (63.0) |
| Father ever international migrant |  |  |  | -83.4 | -96.2 |
|  |  |  |  | (259.8) | (285.2) |
| Any brother international migrant |  |  |  | 119.2 | 213.7 |
|  |  |  |  | (178.3) | (157.9) |
| Number of households in a bari in 82 census |  |  |  |  | -1.6 |
|  |  |  |  |  | (10.5) |
| Family size-82 |  |  |  |  | 3.5 |
|  |  |  |  |  | (24.1) |
| is wall made of tin or better materials? |  |  |  |  | 474.9 |
|  |  |  |  |  | (440.2) |
| wall made of tin or tin mix in 1982 |  |  |  |  | 251.5 |
|  |  |  |  |  | (196.6) |
| is roof made of tin or better materials? |  |  |  |  | 11.9 |
|  |  |  |  |  | (102.0) |
| number of boats owned-82 |  |  |  |  | -71.2 |
|  |  |  |  |  | (85.1) |
| number of rooms in the HH-82 |  |  |  |  | -145.9 |
|  |  |  |  |  | (121.2) |
| ch_hhc_asset82 |  |  |  |  | -24.6 |
|  |  |  |  |  | (43.5) |
| number of cows owned-82 |  |  |  |  | 8.1 |
|  |  |  |  |  | (54.5) |
| use any fixed latrine? |  |  |  |  | -630.2 |
|  |  |  |  |  | (244.0)** |
| HH uses tubewell water for drinking 1982 |  |  |  |  | 98.1 |
|  |  |  |  |  | (156.3) |
| HH uses river or ditch/canal water for drinking in 1982 |  |  |  |  | 227.5 |
|  |  |  |  |  | (192.9) |
| Head of HH's Years of Education, 82, Maktab=0 |  |  |  |  | 0.9 |
|  |  |  |  |  | (21.2) |
| Head of HH does Agriculture (=1 if Y, =0 if N) |  |  |  |  | -7.3 |
|  |  |  |  |  | (137.2) |
| Head of HH does Fishing (=1 if Y, =0 if N) |  |  |  |  | 177.6 |
|  |  |  |  |  | (142.5) |
| Head of HH's age in 1982 Census (=. if not head) |  |  |  |  | 1.8 |
|  |  |  |  |  | (3.4) |
| receive any remittance? |  |  |  |  | -64.1 |
|  |  |  |  |  | (119.5) |
| Constant | 4,453.7 | 786.3 | 23.9 | 44.8 | 467.3 |
|  | (203.0)*** | (1,440.5) | (1,488.0) | (1,489.0) | (1,596.7) |
|  |  |  |  |  |  |
| Observations | 4,296 | 4,296 | 4,296 | 4,296 | 3,792 |
| R-squared | 0.2 | 0.2 | 0.2 | 0.2 | 0.2 |
| F test statistics | 59.51 | 46.07 | 29.60 | 28.39 | 19.50 |
| Degrees of freedom | 9 | 16 | 27 | 29 | 46 |
| Robust standard errors in parentheses, *** p<0.001, ** p<0.01, * p<0.05, + p<0.1 | | | | | |

| Full models, OLS regression of hours worked | | | | | |
| --- | --- | --- | --- | --- | --- |
| VARIABLES | (1) | (2) | (3) | (4) | (5) |
|  |  |  |  |  |  |
| m2_mig_status2 = 0, Non-migrant | -903.7 | -910.3 | -926.6 | -947.8 | -1,013.1 |
|  | (63.8)*** | (67.5)*** | (67.4)*** | (68.2)*** | (72.9)*** |
| m2_mig_status2 = 1, Internal migrant | -335.7 | -270.1 | -282.4 | -312.5 | -327.5 |
|  | (68.0)*** | (68.4)*** | (69.2)*** | (71.6)*** | (77.2)*** |
| m2_mig_status2 = 3, Return int'l migrant | -1,019.7 | -967.2 | -955.6 | -955.3 | -991.1 |
|  | (144.0)*** | (146.4)*** | (142.6)*** | (140.9)*** | (153.3)*** |
| Age 25-29 | 498.6 | 466.4 | 464.3 | 453.4 | 441.7 |
|  | (98.9)*** | (94.5)*** | (92.6)*** | (91.6)*** | (95.2)*** |
| Age 30-34 | 730.6 | 735.1 | 717.1 | 696.4 | 703.4 |
|  | (94.6)*** | (92.2)*** | (91.8)*** | (91.1)*** | (98.3)*** |
| Age 35-39 | 637.3 | 680.0 | 651.1 | 623.2 | 614.6 |
|  | (105.2)*** | (104.3)*** | (105.4)*** | (105.4)*** | (112.9)*** |
| Age 40-44 | 850.2 | 833.3 | 808.4 | 776.1 | 815.5 |
|  | (111.9)*** | (112.8)*** | (117.0)*** | (116.5)*** | (120.7)*** |
| Age 45-49 | 614.2 | 581.2 | 559.4 | 527.0 | 547.4 |
|  | (118.4)*** | (118.0)*** | (121.4)*** | (121.0)*** | (125.8)*** |
| Age 50+ | 470.5 | 408.1 | 384.5 | 349.3 | 334.9 |
|  | (126.0)*** | (126.1)** | (132.2)** | (131.4)** | (139.7)* |
| Hindu religion |  | 269.3 | 246.0 | 234.8 | 226.2 |
|  |  | (80.0)*** | (80.9)** | (80.1)** | (90.8)* |
| Schooling - 1-4 years |  | 122.9 | 128.6 | 124.8 | 117.4 |
|  |  | (94.6) | (94.7) | (94.2) | (99.7) |
| Schooling - 5-9 years |  | 155.7 | 208.4 | 207.7 | 199.6 |
|  |  | (90.0)+ | (92.2)* | (91.9)* | (100.0)* |
| Schooling - Lower Secondary |  | -169.4 | -64.9 | -52.7 | -56.6 |
|  |  | (110.0) | (115.5) | (114.8) | (127.5) |
| Schooling - Upper Secondarry |  | -649.7 | -510.2 | -493.9 | -475.7 |
|  |  | (129.7)*** | (137.6)*** | (138.1)*** | (147.8)** |
| Schooling - College |  | -446.6 | -264.5 | -268.4 | -253.3 |
|  |  | (133.7)*** | (145.2)+ | (144.3)+ | (153.5)+ |
| Height (cm) |  | 1.6 | 3.1 | 3.1 | 2.5 |
|  |  | (4.6) | (4.5) | (4.5) | (4.8) |
| Household asssets, 1996 (logged) |  |  | -42.9 | -35.3 | -43.7 |
|  |  |  | (21.8)* | (21.6) | (24.3)+ |
| Mother's schooling = 1, 1-4 years |  |  | -153.7 | -156.1 | -157.0 |
|  |  |  | (76.8)* | (76.3)* | (79.5)* |
| Mother's schooling = 2, 5-9 years |  |  | -227.6 | -200.7 | -204.4 |
|  |  |  | (98.9)* | (99.3)* | (105.6)+ |
| Mother's schooling = 3, 10+ years |  |  | -766.1 | -759.7 | -750.8 |
|  |  |  | (294.9)** | (291.7)** | (321.4)* |
| Father's schooling = 1, 1-4 years |  |  | 7.0 | 10.1 | -1.2 |
|  |  |  | (66.5) | (66.4) | (70.4) |
| Father's schooling = 2, 5-9 years |  |  | 107.9 | 119.2 | 79.9 |
|  |  |  | (75.4) | (75.2) | (81.9) |
| Father's schooling = 3, 10+ years |  |  | -17.7 | -21.8 | -77.0 |
|  |  |  | (116.8) | (116.9) | (133.3) |
| Younger brothers |  |  | 34.4 | 40.1 | 43.3 |
|  |  |  | (26.3) | (27.0) | (28.1) |
| Older brothers |  |  | -11.4 | -10.1 | -9.5 |
|  |  |  | (22.8) | (23.6) | (26.2) |
| Younger sisters |  |  | -23.7 | -24.3 | -19.2 |
|  |  |  | (27.8) | (27.5) | (29.0) |
| Older sisters |  |  | 1.6 | -2.6 | -1.0 |
|  |  |  | (24.6) | (24.5) | (26.4) |
| Father ever international migrant |  |  |  | -379.2 | -381.9 |
|  |  |  |  | (121.9)** | (129.8)** |
| Any brother international migrant |  |  |  | -84.2 | -47.0 |
|  |  |  |  | (64.4) | (67.3) |
| Number of households in a bari in 82 census |  |  |  |  | -10.8 |
|  |  |  |  |  | (5.5)+ |
| Family size-82 |  |  |  |  | -9.7 |
|  |  |  |  |  | (12.7) |
| is wall made of tin or better materials? |  |  |  |  | -209.0 |
|  |  |  |  |  | (114.2)+ |
| wall made of tin or tin mix in 1982 |  |  |  |  | 112.4 |
|  |  |  |  |  | (73.6) |
| is roof made of tin or better materials? |  |  |  |  | 53.0 |
|  |  |  |  |  | (83.9) |
| number of boats owned-82 |  |  |  |  | -28.9 |
|  |  |  |  |  | (49.2) |
| number of rooms in the HH-82 |  |  |  |  | 49.6 |
|  |  |  |  |  | (61.7) |
| ch_hhc_asset82 |  |  |  |  | 29.5 |
|  |  |  |  |  | (25.8) |
| number of cows owned-82 |  |  |  |  | -40.9 |
|  |  |  |  |  | (20.6)* |
| use any fixed latrine? |  |  |  |  | -245.0 |
|  |  |  |  |  | (143.7)+ |
| HH uses tubewell water for drinking 1982 |  |  |  |  | 107.1 |
|  |  |  |  |  | (76.4) |
| HH uses river or ditch/canal water for drinking in 1982 |  |  |  |  | 16.9 |
|  |  |  |  |  | (83.3) |
| Head of HH's Years of Education, 82, Maktab=0 |  |  |  |  | -2.8 |
|  |  |  |  |  | (11.4) |
| Head of HH does Agriculture (=1 if Y, =0 if N) |  |  |  |  | -68.2 |
|  |  |  |  |  | (66.7) |
| Head of HH does Fishing (=1 if Y, =0 if N) |  |  |  |  | -112.3 |
|  |  |  |  |  | (116.0) |
| Head of HH's age in 1982 Census (=. if not head) |  |  |  |  | 0.4 |
|  |  |  |  |  | (2.0) |
| receive any remittance? |  |  |  |  | -78.2 |
|  |  |  |  |  | (70.8) |
| Constant | 2,941.2 | 2,667.8 | 2,918.0 | 2,903.4 | 3,422.2 |
|  | (87.6)*** | (751.0)*** | (760.3)*** | (758.2)*** | (832.2)*** |
|  |  |  |  |  |  |
| Observations | 4,296 | 4,296 | 4,296 | 4,296 | 3,792 |
| R-squared | 0.1 | 0.2 | 0.2 | 0.2 | 0.2 |
| F test statistics | 35.35 | 25.29 | 17.80 | 17.15 | 11.82 |
| Degrees of freedom | 9 | 16 | 27 | 29 | 46 |
| Robust standard errors in parentheses, *** p<0.001, ** p<0.01, * p<0.05, + p<0.1 | | | | | |

| Full models, OLS regression of hourly wage in US$ | | | | | |
| --- | --- | --- | --- | --- | --- |
| VARIABLES | (1) | (2) | (3) | (4) | (5) |
|  |  |  |  |  |  |
| m2_mig_status2 = 0, Non-migrant | -1.034 | -0.994 | -0.985 | -0.981 | -0.952 |
|  | (0.067)*** | (0.065)*** | (0.066)*** | (0.065)*** | (0.071)*** |
| m2_mig_status2 = 1, Internal migrant | -0.830 | -0.874 | -0.860 | -0.855 | -0.804 |
|  | (0.066)*** | (0.068)*** | (0.069)*** | (0.071)*** | (0.075)*** |
| m2_mig_status2 = 3, Return int'l migrant | -0.821 | -0.849 | -0.854 | -0.854 | -0.853 |
|  | (0.103)*** | (0.095)*** | (0.096)*** | (0.097)*** | (0.105)*** |
| Age 25-29 | 0.143 | 0.135 | 0.125 | 0.126 | 0.126 |
|  | (0.059)* | (0.058)* | (0.057)* | (0.058)* | (0.060)* |
| Age 30-34 | 0.223 | 0.171 | 0.149 | 0.151 | 0.176 |
|  | (0.068)** | (0.066)** | (0.067)* | (0.067)* | (0.076)* |
| Age 35-39 | 0.181 | 0.095 | 0.061 | 0.065 | 0.079 |
|  | (0.056)** | (0.063) | (0.063) | (0.063) | (0.070) |
| Age 40-44 | 0.175 | 0.152 | 0.107 | 0.111 | 0.130 |
|  | (0.081)* | (0.083)+ | (0.078) | (0.077) | (0.078)+ |
| Age 45-49 | 0.113 | 0.141 | 0.087 | 0.091 | 0.052 |
|  | (0.066)+ | (0.072)+ | (0.078) | (0.078) | (0.081) |
| Age 50+ | 0.099 | 0.134 | 0.077 | 0.082 | 0.089 |
|  | (0.075) | (0.080)+ | (0.084) | (0.083) | (0.093) |
| Hindu religion |  | -0.044 | -0.035 | -0.033 | -0.056 |
|  |  | (0.046) | (0.046) | (0.046) | (0.059) |
| Schooling - 1-4 years |  | -0.004 | -0.020 | -0.019 | -0.005 |
|  |  | (0.037) | (0.038) | (0.038) | (0.038) |
| Schooling - 5-9 years |  | 0.083 | 0.040 | 0.040 | 0.068 |
|  |  | (0.045)+ | (0.048) | (0.047) | (0.051) |
| Schooling - Lower Secondary |  | 0.244 | 0.178 | 0.176 | 0.193 |
|  |  | (0.095)* | (0.096)+ | (0.095)+ | (0.102)+ |
| Schooling - Upper Secondarry |  | 0.404 | 0.325 | 0.321 | 0.298 |
|  |  | (0.091)*** | (0.102)** | (0.103)** | (0.107)** |
| Schooling - College |  | 0.826 | 0.733 | 0.733 | 0.630 |
|  |  | (0.149)*** | (0.165)*** | (0.165)*** | (0.165)*** |
| Height (cm) |  | 0.008 | 0.007 | 0.007 | 0.009 |
|  |  | (0.003)** | (0.003)* | (0.003)* | (0.003)** |
| Household asssets, 1996 (logged) |  |  | 0.041 | 0.040 | 0.031 |
|  |  |  | (0.015)** | (0.015)** | (0.017)+ |
| Mother's schooling = 1, 1-4 years |  |  | -0.013 | -0.013 | -0.040 |
|  |  |  | (0.058) | (0.058) | (0.058) |
| Mother's schooling = 2, 5-9 years |  |  | -0.061 | -0.066 | -0.060 |
|  |  |  | (0.072) | (0.073) | (0.079) |
| Mother's schooling = 3, 10+ years |  |  | 0.044 | 0.045 | -0.373 |
|  |  |  | (0.419) | (0.419) | (0.260) |
| Father's schooling = 1, 1-4 years |  |  | 0.022 | 0.021 | 0.034 |
|  |  |  | (0.053) | (0.054) | (0.059) |
| Father's schooling = 2, 5-9 years |  |  | 0.048 | 0.046 | 0.034 |
|  |  |  | (0.055) | (0.055) | (0.055) |
| Father's schooling = 3, 10+ years |  |  | 0.076 | 0.076 | 0.069 |
|  |  |  | (0.105) | (0.105) | (0.124) |
| Younger brothers |  |  | 0.006 | 0.005 | 0.002 |
|  |  |  | (0.017) | (0.017) | (0.018) |
| Older brothers |  |  | 0.023 | 0.023 | -0.001 |
|  |  |  | (0.026) | (0.028) | (0.021) |
| Younger sisters |  |  | 0.003 | 0.003 | -0.005 |
|  |  |  | (0.017) | (0.017) | (0.019) |
| Older sisters |  |  | -0.032 | -0.032 | -0.038 |
|  |  |  | (0.018)+ | (0.018)+ | (0.021)+ |
| Father ever international migrant |  |  |  | 0.068 | 0.078 |
|  |  |  |  | (0.102) | (0.114) |
| Any brother international migrant |  |  |  | 0.015 | 0.037 |
|  |  |  |  | (0.053) | (0.051) |
| Number of households in a bari in 82 census |  |  |  |  | -0.001 |
|  |  |  |  |  | (0.003) |
| Family size-82 |  |  |  |  | 0.005 |
|  |  |  |  |  | (0.011) |
| is wall made of tin or better materials? |  |  |  |  | 0.163 |
|  |  |  |  |  | (0.126) |
| wall made of tin or tin mix in 1982 |  |  |  |  | 0.061 |
|  |  |  |  |  | (0.073) |
| is roof made of tin or better materials? |  |  |  |  | -0.033 |
|  |  |  |  |  | (0.046) |
| number of boats owned-82 |  |  |  |  | -0.049 |
|  |  |  |  |  | (0.045) |
| number of rooms in the HH-82 |  |  |  |  | -0.046 |
|  |  |  |  |  | (0.041) |
| ch_hhc_asset82 |  |  |  |  | -0.013 |
|  |  |  |  |  | (0.019) |
| number of cows owned-82 |  |  |  |  | 0.018 |
|  |  |  |  |  | (0.020) |
| use any fixed latrine? |  |  |  |  | -0.175 |
|  |  |  |  |  | (0.085)* |
| HH uses tubewell water for drinking 1982 |  |  |  |  | 0.047 |
|  |  |  |  |  | (0.053) |
| HH uses river or ditch/canal water for drinking in 1982 |  |  |  |  | 0.097 |
|  |  |  |  |  | (0.064) |
| Head of HH's Years of Education, 82, Maktab=0 |  |  |  |  | 0.003 |
|  |  |  |  |  | (0.008) |
| Head of HH does Agriculture (=1 if Y, =0 if N) |  |  |  |  | 0.018 |
|  |  |  |  |  | (0.046) |
| Head of HH does Fishing (=1 if Y, =0 if N) |  |  |  |  | 0.092 |
|  |  |  |  |  | (0.060) |
| Head of HH's age in 1982 Census (=. if not head) |  |  |  |  | 0.002 |
|  |  |  |  |  | (0.001) |
| receive any remittance? |  |  |  |  | 0.020 |
|  |  |  |  |  | (0.059) |
| Constant | 1.367 | -0.105 | -0.374 | -0.376 | -0.456 |
|  | (0.068)*** | (0.468) | (0.500) | (0.501) | (0.567) |
|  |  |  |  |  |  |
| Observations | 4,047 | 4,047 | 4,047 | 4,047 | 3,565 |
| R-squared | 0.120 | 0.155 | 0.159 | 0.159 | 0.165 |
| F test statistics | 40.26 | 33.14 | 22.28 | 20.98 | 13.39 |
| Degrees of freedom | 9 | 16 | 27 | 29 | 46 |
| Robust standard errors in parentheses, *** p<0.001, ** p<0.01, * p<0.05, + p<0.1 | | | | | |

| Full models, logistic regression of fair/poor self-rated health | | | | | |
| --- | --- | --- | --- | --- | --- |
| VARIABLES | (1) | (2) | (3) | (4) | (5) |
|  |  |  |  |  |  |
| m2_mig_status2 = 0, Non-migrant | 1.240 | 1.112 | 1.091 | 1.088 | 1.077 |
|  | (0.277)*** | (0.289)*** | (0.287)*** | (0.286)*** | (0.301)*** |
| m2_mig_status2 = 1, Internal migrant | 0.830 | 0.755 | 0.759 | 0.755 | 0.702 |
|  | (0.287)** | (0.304)* | (0.304)* | (0.301)* | (0.322)* |
| m2_mig_status2 = 3, Return int'l migrant | 0.918 | 0.886 | 0.855 | 0.854 | 0.767 |
|  | (0.388)* | (0.393)* | (0.394)* | (0.394)* | (0.428)+ |
| Age 25-29 | 0.206 | 0.109 | 0.140 | 0.138 | 0.177 |
|  | (0.253) | (0.257) | (0.260) | (0.260) | (0.280) |
| Age 30-34 | 0.511 | 0.446 | 0.493 | 0.489 | 0.431 |
|  | (0.235)* | (0.242)+ | (0.249)* | (0.250)+ | (0.287) |
| Age 35-39 | 0.621 | 0.559 | 0.629 | 0.624 | 0.648 |
|  | (0.255)* | (0.253)* | (0.259)* | (0.261)* | (0.283)* |
| Age 40-44 | 1.110 | 0.939 | 0.976 | 0.970 | 0.879 |
|  | (0.262)*** | (0.265)*** | (0.282)*** | (0.286)*** | (0.297)** |
| Age 45-49 | 1.342 | 1.112 | 1.144 | 1.138 | 1.117 |
|  | (0.256)*** | (0.265)*** | (0.279)*** | (0.283)*** | (0.296)*** |
| Age 50+ | 2.013 | 1.839 | 1.824 | 1.818 | 1.816 |
|  | (0.243)*** | (0.249)*** | (0.262)*** | (0.265)*** | (0.290)*** |
| Hindu religion |  | -0.599 | -0.631 | -0.631 | -0.522 |
|  |  | (0.221)** | (0.223)** | (0.223)** | (0.259)* |
| Schooling - 1-4 years |  | -0.004 | 0.000 | -0.000 | -0.032 |
|  |  | (0.193) | (0.196) | (0.196) | (0.208) |
| Schooling - 5-9 years |  | -0.490 | -0.479 | -0.479 | -0.521 |
|  |  | (0.193)* | (0.200)* | (0.200)* | (0.219)* |
| Schooling - Lower Secondary |  | -0.710 | -0.684 | -0.683 | -0.640 |
|  |  | (0.266)** | (0.290)* | (0.290)* | (0.328)+ |
| Schooling - Upper Secondarry |  | -0.762 | -0.712 | -0.709 | -0.981 |
|  |  | (0.267)** | (0.288)* | (0.287)* | (0.327)** |
| Schooling - College |  | -0.478 | -0.442 | -0.443 | -0.404 |
|  |  | (0.333) | (0.355) | (0.354) | (0.386) |
| Height (cm) |  | -0.018 | -0.019 | -0.019 | -0.019 |
|  |  | (0.011)+ | (0.011)+ | (0.011)+ | (0.012) |
| Household asssets, 1996 (logged) |  |  | 0.021 | 0.022 | 0.019 |
|  |  |  | (0.053) | (0.052) | (0.060) |
| Mother's schooling = 1, 1-4 years |  |  | -0.086 | -0.087 | -0.068 |
|  |  |  | (0.203) | (0.203) | (0.214) |
| Mother's schooling = 2, 5-9 years |  |  | -0.279 | -0.275 | -0.283 |
|  |  |  | (0.276) | (0.274) | (0.298) |
| Mother's schooling = 3, 10+ years |  |  | 0.182 | 0.186 | 0.203 |
|  |  |  | (0.734) | (0.733) | (0.754) |
| Father's schooling = 1, 1-4 years |  |  | -0.348 | -0.348 | -0.372 |
|  |  |  | (0.202)+ | (0.202)+ | (0.219)+ |
| Father's schooling = 2, 5-9 years |  |  | -0.089 | -0.087 | -0.034 |
|  |  |  | (0.190) | (0.191) | (0.212) |
| Father's schooling = 3, 10+ years |  |  | 0.033 | 0.033 | 0.140 |
|  |  |  | (0.291) | (0.291) | (0.358) |
| Younger brothers |  |  | -0.157 | -0.157 | -0.195 |
|  |  |  | (0.072)* | (0.074)* | (0.076)* |
| Older brothers |  |  | -0.036 | -0.036 | -0.034 |
|  |  |  | (0.063) | (0.065) | (0.073) |
| Younger sisters |  |  | -0.003 | -0.003 | 0.042 |
|  |  |  | (0.063) | (0.063) | (0.067) |
| Older sisters |  |  | -0.077 | -0.078 | -0.085 |
|  |  |  | (0.081) | (0.081) | (0.087) |
| Father ever international migrant |  |  |  | -0.070 | -0.006 |
|  |  |  |  | (0.341) | (0.360) |
| Any brother international migrant |  |  |  | -0.008 | -0.056 |
|  |  |  |  | (0.156) | (0.169) |
| Number of households in a bari in 82 census |  |  |  |  | 0.020 |
|  |  |  |  |  | (0.013) |
| Family size-82 |  |  |  |  | 0.021 |
|  |  |  |  |  | (0.034) |
| is wall made of tin or better materials? |  |  |  |  | -0.012 |
|  |  |  |  |  | (0.352) |
| wall made of tin or tin mix in 1982 |  |  |  |  | -0.136 |
|  |  |  |  |  | (0.197) |
| is roof made of tin or better materials? |  |  |  |  | 0.001 |
|  |  |  |  |  | (0.214) |
| number of boats owned-82 |  |  |  |  | 0.082 |
|  |  |  |  |  | (0.129) |
| number of rooms in the HH-82 |  |  |  |  | -0.173 |
|  |  |  |  |  | (0.153) |
| ch_hhc_asset82 |  |  |  |  | -0.098 |
|  |  |  |  |  | (0.066) |
| number of cows owned-82 |  |  |  |  | 0.065 |
|  |  |  |  |  | (0.050) |
| use any fixed latrine? |  |  |  |  | 0.265 |
|  |  |  |  |  | (0.396) |
| HH uses tubewell water for drinking 1982 |  |  |  |  | -0.071 |
|  |  |  |  |  | (0.211) |
| HH uses river or ditch/canal water for drinking in 1982 |  |  |  |  | -0.240 |
|  |  |  |  |  | (0.236) |
| Head of HH's Years of Education, 82, Maktab=0 |  |  |  |  | 0.011 |
|  |  |  |  |  | (0.031) |
| Head of HH does Agriculture (=1 if Y, =0 if N) |  |  |  |  | 0.234 |
|  |  |  |  |  | (0.169) |
| Head of HH does Fishing (=1 if Y, =0 if N) |  |  |  |  | 0.183 |
|  |  |  |  |  | (0.355) |
| Head of HH's age in 1982 Census (=. if not head) |  |  |  |  | 0.008 |
|  |  |  |  |  | (0.005) |
| receive any remittance? |  |  |  |  | 0.309 |
|  |  |  |  |  | (0.174)+ |
| Constant | -3.560 | -0.025 | 0.158 | 0.157 | -0.522 |
|  | (0.312)*** | (1.813) | (1.818) | (1.818) | (1.997) |
|  |  |  |  |  |  |
| Observations | 4,296 | 4,296 | 4,296 | 4,296 | 3,792 |
| Pseudo R-squared | 0.0969 | 0.115 | 0.121 | 0.121 | 0.140 |
| Likelihood ratio chi-square | 164.3 | 195 | 206.5 | 209 | 212.7 |
| Degrees of freedom | 9 | 16 | 27 | 29 | 46 |
| Robust standard errors in parentheses, *** p<0.001, ** p<0.01, * p<0.05, + p<0.1 | | | | | |

| Full models, logistic regression of injury in last 12 months | | | | | |
| --- | --- | --- | --- | --- | --- |
| VARIABLES | (1) | (2) | (3) | (4) | (5) |
|  |  |  |  |  |  |
| m2_mig_status2 = 0, Non-migrant | 0.524 | 0.462 | 0.433 | 0.475 | 0.386 |
|  | (0.255)* | (0.273)+ | (0.274) | (0.275)+ | (0.289) |
| m2_mig_status2 = 1, Internal migrant | -0.155 | -0.196 | -0.236 | -0.172 | -0.234 |
|  | (0.292) | (0.316) | (0.315) | (0.320) | (0.348) |
| m2_mig_status2 = 3, Return int'l migrant | 0.451 | 0.429 | 0.417 | 0.398 | 0.354 |
|  | (0.396) | (0.405) | (0.406) | (0.410) | (0.444) |
| Age 25-29 | -0.091 | -0.150 | -0.116 | -0.127 | -0.164 |
|  | (0.257) | (0.258) | (0.262) | (0.264) | (0.291) |
| Age 30-34 | 0.080 | 0.039 | 0.095 | 0.058 | 0.174 |
|  | (0.261) | (0.273) | (0.283) | (0.283) | (0.313) |
| Age 35-39 | 0.002 | -0.038 | 0.034 | -0.008 | 0.217 |
|  | (0.276) | (0.262) | (0.267) | (0.270) | (0.296) |
| Age 40-44 | -0.202 | -0.321 | -0.253 | -0.306 | -0.183 |
|  | (0.373) | (0.370) | (0.397) | (0.402) | (0.427) |
| Age 45-49 | -0.000 | -0.157 | -0.103 | -0.152 | -0.051 |
|  | (0.335) | (0.337) | (0.343) | (0.347) | (0.353) |
| Age 50+ | -0.159 | -0.290 | -0.271 | -0.319 | -0.281 |
|  | (0.361) | (0.367) | (0.385) | (0.388) | (0.427) |
| Hindu religion |  | -0.525 | -0.572 | -0.564 | -0.558 |
|  |  | (0.281)+ | (0.284)* | (0.285)* | (0.381) |
| Schooling - 1-4 years |  | 0.029 | 0.054 | 0.046 | -0.133 |
|  |  | (0.272) | (0.270) | (0.269) | (0.295) |
| Schooling - 5-9 years |  | -0.283 | -0.203 | -0.215 | -0.422 |
|  |  | (0.250) | (0.246) | (0.247) | (0.278) |
| Schooling - Lower Secondary |  | -0.406 | -0.294 | -0.307 | -0.566 |
|  |  | (0.323) | (0.336) | (0.335) | (0.372) |
| Schooling - Upper Secondarry |  | -0.606 | -0.466 | -0.503 | -0.689 |
|  |  | (0.363)+ | (0.408) | (0.416) | (0.443) |
| Schooling - College |  | -0.378 | -0.198 | -0.203 | -0.377 |
|  |  | (0.460) | (0.481) | (0.481) | (0.511) |
| Height (cm) |  | -0.010 | -0.008 | -0.008 | -0.003 |
|  |  | (0.013) | (0.014) | (0.014) | (0.014) |
| Household asssets, 1996 (logged) |  |  | -0.087 | -0.104 | -0.150 |
|  |  |  | (0.067) | (0.067) | (0.078)+ |
| Mother's schooling = 1, 1-4 years |  |  | -0.140 | -0.146 | -0.145 |
|  |  |  | (0.234) | (0.233) | (0.252) |
| Mother's schooling = 2, 5-9 years |  |  | 0.072 | 0.078 | 0.204 |
|  |  |  | (0.325) | (0.327) | (0.361) |
| Mother's schooling = 3, 10+ years |  |  | -0.283 | -0.256 | 0.152 |
|  |  |  | (0.714) | (0.725) | (0.815) |
| Father's schooling = 1, 1-4 years |  |  | -0.359 | -0.339 | -0.452 |
|  |  |  | (0.233) | (0.234) | (0.258)+ |
| Father's schooling = 2, 5-9 years |  |  | 0.095 | 0.093 | 0.130 |
|  |  |  | (0.247) | (0.248) | (0.288) |
| Father's schooling = 3, 10+ years |  |  | -0.175 | -0.171 | -0.300 |
|  |  |  | (0.352) | (0.354) | (0.493) |
| Younger brothers |  |  | -0.104 | -0.138 | -0.116 |
|  |  |  | (0.081) | (0.083)+ | (0.093) |
| Older brothers |  |  | 0.004 | -0.035 | 0.023 |
|  |  |  | (0.089) | (0.089) | (0.095) |
| Younger sisters |  |  | 0.064 | 0.068 | 0.081 |
|  |  |  | (0.080) | (0.080) | (0.090) |
| Older sisters |  |  | -0.059 | -0.066 | -0.048 |
|  |  |  | (0.092) | (0.093) | (0.101) |
| Father ever international migrant |  |  |  | -0.082 | 0.120 |
|  |  |  |  | (0.352) | (0.369) |
| Any brother international migrant |  |  |  | 0.326 | 0.344 |
|  |  |  |  | (0.186)+ | (0.197)+ |
| Number of households in a bari in 82 census |  |  |  |  | 0.011 |
|  |  |  |  |  | (0.017) |
| Family size-82 |  |  |  |  | -0.013 |
|  |  |  |  |  | (0.038) |
| is wall made of tin or better materials? |  |  |  |  | 0.112 |
|  |  |  |  |  | (0.444) |
| wall made of tin or tin mix in 1982 |  |  |  |  | -0.423 |
|  |  |  |  |  | (0.244)+ |
| is roof made of tin or better materials? |  |  |  |  | 0.151 |
|  |  |  |  |  | (0.254) |
| number of boats owned-82 |  |  |  |  | -0.174 |
|  |  |  |  |  | (0.166) |
| number of rooms in the HH-82 |  |  |  |  | 0.105 |
|  |  |  |  |  | (0.174) |
| ch_hhc_asset82 |  |  |  |  | -0.072 |
|  |  |  |  |  | (0.081) |
| number of cows owned-82 |  |  |  |  | 0.075 |
|  |  |  |  |  | (0.062) |
| use any fixed latrine? |  |  |  |  | 0.687 |
|  |  |  |  |  | (0.670) |
| HH uses tubewell water for drinking 1982 |  |  |  |  | 0.127 |
|  |  |  |  |  | (0.277) |
| HH uses river or ditch/canal water for drinking in 1982 |  |  |  |  | 0.428 |
|  |  |  |  |  | (0.294) |
| Head of HH's Years of Education, 82, Maktab=0 |  |  |  |  | 0.058 |
|  |  |  |  |  | (0.041) |
| Head of HH does Agriculture (=1 if Y, =0 if N) |  |  |  |  | 0.200 |
|  |  |  |  |  | (0.202) |
| Head of HH does Fishing (=1 if Y, =0 if N) |  |  |  |  | -0.042 |
|  |  |  |  |  | (0.429) |
| Head of HH's age in 1982 Census (=. if not head) |  |  |  |  | 0.000 |
|  |  |  |  |  | (0.006) |
| receive any remittance? |  |  |  |  | 0.287 |
|  |  |  |  |  | (0.208) |
| Constant | -2.750 | -0.801 | -0.009 | 0.062 | -1.427 |
|  | (0.292)*** | (2.204) | (2.299) | (2.309) | (2.572) |
|  |  |  |  |  |  |
| Observations | 4,296 | 4,296 | 4,296 | 4,296 | 3,792 |
| Pseudo R-squared | 0.0117 | 0.0203 | 0.0275 | 0.0299 | 0.0491 |
| Likelihood ratio chi-square | 17.39 | 30.18 | 40.30 | 43.46 | 71.58 |
| Degrees of freedom | 9 | 16 | 27 | 29 | 46 |
| Robust standard errors in parentheses, *** p<0.001, ** p<0.01, * p<0.05, + p<0.1 | | | | | |

| Full models, logistic regression of work-related injury in last 12 months | | | | | |
| --- | --- | --- | --- | --- | --- |
| VARIABLES | (1) | (2) | (3) | (4) | (5) |
|  |  |  |  |  |  |
| m2_mig_status2 = 0, Non-migrant | 0.084 | -0.074 | -0.081 | -0.013 | -0.046 |
|  | (0.292) | (0.308) | (0.308) | (0.312) | (0.326) |
| m2_mig_status2 = 1, Internal migrant | -0.401 | -0.517 | -0.517 | -0.415 | -0.386 |
|  | (0.343) | (0.376) | (0.370) | (0.383) | (0.400) |
| m2_mig_status2 = 3, Return int'l migrant | 0.224 | 0.173 | 0.189 | 0.166 | 0.132 |
|  | (0.479) | (0.492) | (0.491) | (0.499) | (0.549) |
| Age 25-29 | -0.322 | -0.433 | -0.428 | -0.445 | -0.436 |
|  | (0.320) | (0.324) | (0.327) | (0.331) | (0.371) |
| Age 30-34 | 0.098 | 0.005 | 0.015 | -0.026 | 0.148 |
|  | (0.328) | (0.345) | (0.351) | (0.356) | (0.389) |
| Age 35-39 | 0.029 | -0.084 | -0.077 | -0.124 | 0.125 |
|  | (0.357) | (0.334) | (0.337) | (0.343) | (0.375) |
| Age 40-44 | -0.626 | -0.836 | -0.885 | -0.941 | -0.769 |
|  | (0.459) | (0.457)+ | (0.479)+ | (0.481)+ | (0.514) |
| Age 45-49 | -0.169 | -0.452 | -0.518 | -0.574 | -0.402 |
|  | (0.388) | (0.393) | (0.399) | (0.403) | (0.432) |
| Age 50+ | 0.241 | -0.001 | -0.138 | -0.186 | 0.039 |
|  | (0.416) | (0.427) | (0.443) | (0.453) | (0.492) |
| Hindu religion |  | -0.445 | -0.494 | -0.487 | -0.602 |
|  |  | (0.348) | (0.349) | (0.351) | (0.457) |
| Schooling - 1-4 years |  | 0.120 | 0.153 | 0.137 | -0.000 |
|  |  | (0.310) | (0.313) | (0.311) | (0.338) |
| Schooling - 5-9 years |  | -0.489 | -0.367 | -0.379 | -0.591 |
|  |  | (0.291)+ | (0.287) | (0.287) | (0.338)+ |
| Schooling - Lower Secondary |  | -0.531 | -0.324 | -0.348 | -0.472 |
|  |  | (0.395) | (0.408) | (0.407) | (0.449) |
| Schooling - Upper Secondarry |  | -1.067 | -0.790 | -0.844 | -1.039 |
|  |  | (0.628)+ | (0.683) | (0.701) | (0.698) |
| Schooling - College |  | -0.304 | 0.014 | 0.009 | -0.115 |
|  |  | (0.596) | (0.600) | (0.598) | (0.573) |
| Height (cm) |  | -0.016 | -0.015 | -0.014 | -0.001 |
|  |  | (0.014) | (0.015) | (0.015) | (0.016) |
| Household asssets, 1996 (logged) |  |  | -0.017 | -0.045 | -0.122 |
|  |  |  | (0.081) | (0.081) | (0.099) |
| Mother's schooling = 1, 1-4 years |  |  | -0.411 | -0.417 | -0.358 |
|  |  |  | (0.273) | (0.272) | (0.299) |
| Mother's schooling = 2, 5-9 years |  |  | -0.321 | -0.323 | -0.168 |
|  |  |  | (0.374) | (0.374) | (0.402) |
| Mother's schooling = 3, 10+ years |  |  | -0.825 | -0.778 | -0.351 |
|  |  |  | (1.160) | (1.172) | (1.180) |
| Father's schooling = 1, 1-4 years |  |  | -0.477 | -0.452 | -0.339 |
|  |  |  | (0.281)+ | (0.281) | (0.303) |
| Father's schooling = 2, 5-9 years |  |  | -0.047 | -0.056 | 0.063 |
|  |  |  | (0.275) | (0.275) | (0.341) |
| Father's schooling = 3, 10+ years |  |  | -0.550 | -0.546 | -0.290 |
|  |  |  | (0.540) | (0.539) | (0.684) |
| Younger brothers |  |  | -0.065 | -0.109 | -0.064 |
|  |  |  | (0.085) | (0.092) | (0.098) |
| Older brothers |  |  | -0.003 | -0.052 | 0.003 |
|  |  |  | (0.105) | (0.105) | (0.108) |
| Younger sisters |  |  | 0.026 | 0.031 | 0.037 |
|  |  |  | (0.095) | (0.096) | (0.107) |
| Older sisters |  |  | -0.117 | -0.123 | -0.080 |
|  |  |  | (0.115) | (0.115) | (0.120) |
| Father ever international migrant |  |  |  | 0.073 | 0.276 |
|  |  |  |  | (0.443) | (0.445) |
| Any brother international migrant |  |  |  | 0.455 | 0.546 |
|  |  |  |  | (0.233)+ | (0.252)* |
| Number of households in a bari in 82 census |  |  |  |  | 0.011 |
|  |  |  |  |  | (0.018) |
| Family size-82 |  |  |  |  | -0.016 |
|  |  |  |  |  | (0.044) |
| is wall made of tin or better materials? |  |  |  |  | 0.530 |
|  |  |  |  |  | (0.476) |
| wall made of tin or tin mix in 1982 |  |  |  |  | -0.389 |
|  |  |  |  |  | (0.302) |
| is roof made of tin or better materials? |  |  |  |  | 0.276 |
|  |  |  |  |  | (0.306) |
| number of boats owned-82 |  |  |  |  | -0.219 |
|  |  |  |  |  | (0.203) |
| number of rooms in the HH-82 |  |  |  |  | 0.023 |
|  |  |  |  |  | (0.190) |
| ch_hhc_asset82 |  |  |  |  | -0.074 |
|  |  |  |  |  | (0.095) |
| number of cows owned-82 |  |  |  |  | 0.091 |
|  |  |  |  |  | (0.078) |
| use any fixed latrine? |  |  |  |  | 1.683 |
|  |  |  |  |  | (1.054) |
| HH uses tubewell water for drinking 1982 |  |  |  |  | -0.033 |
|  |  |  |  |  | (0.365) |
| HH uses river or ditch/canal water for drinking in 1982 |  |  |  |  | 0.595 |
|  |  |  |  |  | (0.366) |
| Head of HH's Years of Education, 82, Maktab=0 |  |  |  |  | 0.018 |
|  |  |  |  |  | (0.051) |
| Head of HH does Agriculture (=1 if Y, =0 if N) |  |  |  |  | 0.361 |
|  |  |  |  |  | (0.255) |
| Head of HH does Fishing (=1 if Y, =0 if N) |  |  |  |  | 0.389 |
|  |  |  |  |  | (0.495) |
| Head of HH's age in 1982 Census (=. if not head) |  |  |  |  | 0.006 |
|  |  |  |  |  | (0.007) |
| receive any remittance? |  |  |  |  | 0.222 |
|  |  |  |  |  | (0.249) |
| Constant | -2.867 | 0.395 | 0.597 | 0.756 | -3.350 |
|  | (0.347)*** | (2.313) | (2.450) | (2.495) | (2.795) |
|  |  |  |  |  |  |
| Observations | 4,296 | 4,296 | 4,296 | 4,296 | 3,792 |
| Pseudo R-squared | 0.0116 | 0.0276 | 0.0374 | 0.0417 | 0.0698 |
| Likelihood ratio chi-square | 10.98 | 28.97 | 40.58 | 44.77 | 88.71 |
| Degrees of freedom | 9 | 16 | 27 | 29 | 46 |
| Robust standard errors in parentheses, *** p<0.001, ** p<0.01, * p<0.05, + p<0.1 | | | | | |

| Full models, logistic regression of current smoker status | | | | | |
| --- | --- | --- | --- | --- | --- |
| VARIABLES | (1) | (2) | (3) | (4) | (5) |
|  |  |  |  |  |  |
| m2_mig_status2 = 0, Non-migrant | 0.366 | 0.258 | 0.231 | 0.224 | 0.184 |
|  | (0.132)** | (0.143)+ | (0.144) | (0.145) | (0.152) |
| m2_mig_status2 = 1, Internal migrant | 0.284 | 0.259 | 0.240 | 0.230 | 0.256 |
|  | (0.140)* | (0.148)+ | (0.150) | (0.152) | (0.160) |
| m2_mig_status2 = 3, Return int'l migrant | 0.242 | 0.235 | 0.236 | 0.237 | 0.290 |
|  | (0.228) | (0.223) | (0.226) | (0.226) | (0.247) |
| Age 25-29 | 0.136 | 0.075 | 0.094 | 0.093 | 0.088 |
|  | (0.150) | (0.153) | (0.154) | (0.153) | (0.163) |
| Age 30-34 | 0.443 | 0.440 | 0.479 | 0.479 | 0.528 |
|  | (0.148)** | (0.152)** | (0.156)** | (0.155)** | (0.168)** |
| Age 35-39 | 0.584 | 0.588 | 0.644 | 0.644 | 0.695 |
|  | (0.161)*** | (0.167)*** | (0.173)*** | (0.173)*** | (0.189)*** |
| Age 40-44 | 0.483 | 0.365 | 0.402 | 0.401 | 0.484 |
|  | (0.180)** | (0.186)* | (0.195)* | (0.197)* | (0.208)* |
| Age 45-49 | 0.663 | 0.463 | 0.487 | 0.486 | 0.597 |
|  | (0.195)*** | (0.200)* | (0.208)* | (0.208)* | (0.214)** |
| Age 50+ | 0.577 | 0.380 | 0.380 | 0.378 | 0.397 |
|  | (0.193)** | (0.201)+ | (0.208)+ | (0.209)+ | (0.226)+ |
| Hindu religion |  | -0.266 | -0.285 | -0.287 | -0.421 |
|  |  | (0.158)+ | (0.161)+ | (0.162)+ | (0.176)* |
| Schooling - 1-4 years |  | -0.093 | -0.095 | -0.095 | -0.047 |
|  |  | (0.156) | (0.156) | (0.156) | (0.164) |
| Schooling - 5-9 years |  | -0.513 | -0.529 | -0.528 | -0.484 |
|  |  | (0.148)*** | (0.151)*** | (0.151)*** | (0.162)** |
| Schooling - Lower Secondary |  | -0.926 | -0.974 | -0.971 | -0.884 |
|  |  | (0.187)*** | (0.198)*** | (0.198)*** | (0.218)*** |
| Schooling - Upper Secondarry |  | -1.042 | -1.112 | -1.107 | -0.977 |
|  |  | (0.216)*** | (0.229)*** | (0.230)*** | (0.242)*** |
| Schooling - College |  | -0.946 | -1.064 | -1.064 | -0.937 |
|  |  | (0.240)*** | (0.262)*** | (0.262)*** | (0.284)*** |
| Height (cm) |  | 0.004 | 0.004 | 0.004 | 0.004 |
|  |  | (0.008) | (0.008) | (0.008) | (0.008) |
| Household asssets, 1996 (logged) |  |  | -0.046 | -0.044 | -0.060 |
|  |  |  | (0.037) | (0.038) | (0.042) |
| Mother's schooling = 1, 1-4 years |  |  | 0.067 | 0.067 | 0.005 |
|  |  |  | (0.128) | (0.128) | (0.139) |
| Mother's schooling = 2, 5-9 years |  |  | 0.147 | 0.151 | 0.180 |
|  |  |  | (0.169) | (0.170) | (0.187) |
| Mother's schooling = 3, 10+ years |  |  | 0.766 | 0.766 | 0.625 |
|  |  |  | (0.438)+ | (0.436)+ | (0.452) |
| Father's schooling = 1, 1-4 years |  |  | -0.102 | -0.103 | -0.058 |
|  |  |  | (0.129) | (0.129) | (0.137) |
| Father's schooling = 2, 5-9 years |  |  | 0.102 | 0.104 | 0.084 |
|  |  |  | (0.131) | (0.132) | (0.149) |
| Father's schooling = 3, 10+ years |  |  | 0.152 | 0.151 | 0.173 |
|  |  |  | (0.195) | (0.196) | (0.232) |
| Younger brothers |  |  | -0.043 | -0.040 | -0.051 |
|  |  |  | (0.046) | (0.047) | (0.051) |
| Older brothers |  |  | -0.088 | -0.085 | -0.081 |
|  |  |  | (0.044)* | (0.045)+ | (0.048)+ |
| Younger sisters |  |  | 0.099 | 0.099 | 0.079 |
|  |  |  | (0.046)* | (0.046)* | (0.050) |
| Older sisters |  |  | 0.015 | 0.015 | 0.016 |
|  |  |  | (0.045) | (0.045) | (0.048) |
| Father ever international migrant |  |  |  | -0.053 | 0.013 |
|  |  |  |  | (0.215) | (0.235) |
| Any brother international migrant |  |  |  | -0.037 | -0.067 |
|  |  |  |  | (0.112) | (0.117) |
| Number of households in a bari in 82 census |  |  |  |  | 0.000 |
|  |  |  |  |  | (0.010) |
| Family size-82 |  |  |  |  | 0.018 |
|  |  |  |  |  | (0.023) |
| is wall made of tin or better materials? |  |  |  |  | 0.265 |
|  |  |  |  |  | (0.201) |
| wall made of tin or tin mix in 1982 |  |  |  |  | -0.041 |
|  |  |  |  |  | (0.135) |
| is roof made of tin or better materials? |  |  |  |  | 0.085 |
|  |  |  |  |  | (0.142) |
| number of boats owned-82 |  |  |  |  | -0.107 |
|  |  |  |  |  | (0.096) |
| number of rooms in the HH-82 |  |  |  |  | 0.005 |
|  |  |  |  |  | (0.101) |
| ch_hhc_asset82 |  |  |  |  | 0.035 |
|  |  |  |  |  | (0.046) |
| number of cows owned-82 |  |  |  |  | 0.013 |
|  |  |  |  |  | (0.035) |
| use any fixed latrine? |  |  |  |  | -0.331 |
|  |  |  |  |  | (0.295) |
| HH uses tubewell water for drinking 1982 |  |  |  |  | 0.159 |
|  |  |  |  |  | (0.154) |
| HH uses river or ditch/canal water for drinking in 1982 |  |  |  |  | 0.263 |
|  |  |  |  |  | (0.166) |
| Head of HH's Years of Education, 82, Maktab=0 |  |  |  |  | -0.017 |
|  |  |  |  |  | (0.020) |
| Head of HH does Agriculture (=1 if Y, =0 if N) |  |  |  |  | -0.155 |
|  |  |  |  |  | (0.113) |
| Head of HH does Fishing (=1 if Y, =0 if N) |  |  |  |  | 0.037 |
|  |  |  |  |  | (0.235) |
| Head of HH's age in 1982 Census (=. if not head) |  |  |  |  | -0.000 |
|  |  |  |  |  | (0.004) |
| receive any remittance? |  |  |  |  | -0.050 |
|  |  |  |  |  | (0.129) |
| Constant | -1.184 | -1.237 | -0.710 | -0.716 | -0.450 |
|  | (0.156)*** | (1.281) | (1.306) | (1.306) | (1.430) |
|  |  |  |  |  |  |
| Observations | 4,284 | 4,284 | 4,284 | 4,284 | 3,783 |
| Pseudo R-squared | 0.0128 | 0.0334 | 0.0407 | 0.0408 | 0.0427 |
| Likelihood ratio chi-square | 37.14 | 93.14 | 114.3 | 114.8 | 109.1 |
| Degrees of freedom | 9 | 16 | 27 | 29 | 46 |
| Robust standard errors in parentheses, *** p<0.001, ** p<0.01, * p<0.05, + p<0.1 | | | | | |

| Full models, OLS regression of Body Mass Index | | | | | |
| --- | --- | --- | --- | --- | --- |
|  | (1) | (2) | (3) | (4) | (5) |
|  |  |  |  |  |  |
| m2_mig_status2 = 0, Non-migrant | -2.809 | -2.516 | -2.464 | -2.403 | -2.384 |
|  | (0.174)*** | (0.188)*** | (0.186)*** | (0.189)*** | (0.199)*** |
| m2_mig_status2 = 1, Internal migrant | -1.508 | -1.452 | -1.404 | -1.304 | -1.224 |
|  | (0.188)*** | (0.197)*** | (0.196)*** | (0.200)*** | (0.216)*** |
| m2_mig_status2 = 3, Return int'l migrant | -0.599 | -0.579 | -0.580 | -0.598 | -0.669 |
|  | (0.337)+ | (0.358) | (0.358) | (0.350)+ | (0.383)+ |
| Age 25-29 | 1.157 | 1.279 | 1.227 | 1.206 | 1.235 |
|  | (0.204)*** | (0.198)*** | (0.199)*** | (0.196)*** | (0.208)*** |
| Age 30-34 | 1.551 | 1.567 | 1.498 | 1.433 | 1.425 |
|  | (0.191)*** | (0.188)*** | (0.195)*** | (0.193)*** | (0.214)*** |
| Age 35-39 | 1.705 | 1.714 | 1.610 | 1.518 | 1.564 |
|  | (0.201)*** | (0.206)*** | (0.215)*** | (0.215)*** | (0.239)*** |
| Age 40-44 | 1.236 | 1.526 | 1.361 | 1.255 | 1.240 |
|  | (0.257)*** | (0.247)*** | (0.255)*** | (0.255)*** | (0.270)*** |
| Age 45-49 | 0.981 | 1.550 | 1.373 | 1.268 | 1.143 |
|  | (0.262)*** | (0.245)*** | (0.252)*** | (0.253)*** | (0.272)*** |
| Age 50+ | 1.422 | 1.972 | 1.787 | 1.702 | 1.683 |
|  | (0.329)*** | (0.326)*** | (0.336)*** | (0.337)*** | (0.361)*** |
| Hindu religion |  | 0.481 | 0.530 | 0.550 | 0.379 |
|  |  | (0.205)* | (0.206)* | (0.207)** | (0.242) |
| Schooling - 1-4 years |  | 0.817 | 0.792 | 0.780 | 0.679 |
|  |  | (0.225)*** | (0.224)*** | (0.225)*** | (0.245)** |
| Schooling - 5-9 years |  | 1.602 | 1.505 | 1.483 | 1.458 |
|  |  | (0.211)*** | (0.217)*** | (0.217)*** | (0.238)*** |
| Schooling - Lower Secondary |  | 2.453 | 2.298 | 2.273 | 2.103 |
|  |  | (0.264)*** | (0.280)*** | (0.277)*** | (0.302)*** |
| Schooling - Upper Secondarry |  | 2.325 | 2.169 | 2.116 | 1.968 |
|  |  | (0.256)*** | (0.275)*** | (0.277)*** | (0.305)*** |
| Schooling - College |  | 2.886 | 2.733 | 2.716 | 2.405 |
|  |  | (0.328)*** | (0.358)*** | (0.358)*** | (0.394)*** |
| Height (cm) |  | 0.003 | 0.001 | 0.002 | 0.001 |
|  |  | (0.012) | (0.012) | (0.012) | (0.013) |
| Household asssets, 1996 (logged) |  |  | 0.149 | 0.126 | 0.122 |
|  |  |  | (0.050)** | (0.051)* | (0.056)* |
| Mother's schooling = 1, 1-4 years |  |  | -0.074 | -0.078 | -0.109 |
|  |  |  | (0.179) | (0.177) | (0.188) |
| Mother's schooling = 2, 5-9 years |  |  | 0.231 | 0.234 | 0.115 |
|  |  |  | (0.223) | (0.222) | (0.242) |
| Mother's schooling = 3, 10+ years |  |  | -0.052 | -0.006 | -0.094 |
|  |  |  | (0.394) | (0.394) | (0.434) |
| Father's schooling = 1, 1-4 years |  |  | 0.116 | 0.150 | 0.073 |
|  |  |  | (0.163) | (0.162) | (0.170) |
| Father's schooling = 2, 5-9 years |  |  | -0.157 | -0.151 | -0.231 |
|  |  |  | (0.178) | (0.177) | (0.196) |
| Father's schooling = 3, 10+ years |  |  | -0.361 | -0.339 | -0.423 |
|  |  |  | (0.256) | (0.255) | (0.294) |
| Younger brothers |  |  | 0.035 | -0.016 | -0.059 |
|  |  |  | (0.067) | (0.069) | (0.074) |
| Older brothers |  |  | 0.018 | -0.047 | -0.051 |
|  |  |  | (0.058) | (0.059) | (0.066) |
| Younger sisters |  |  | 0.062 | 0.067 | 0.102 |
|  |  |  | (0.070) | (0.070) | (0.074) |
| Older sisters |  |  | -0.043 | -0.054 | -0.090 |
|  |  |  | (0.064) | (0.063) | (0.068) |
| Father ever international migrant |  |  |  | -0.183 | -0.142 |
|  |  |  |  | (0.248) | (0.275) |
| Any brother international migrant |  |  |  | 0.543 | 0.518 |
|  |  |  |  | (0.156)*** | (0.162)** |
| Number of households in a bari in 82 census |  |  |  |  | -0.002 |
|  |  |  |  |  | (0.013) |
| Family size-82 |  |  |  |  | 0.023 |
|  |  |  |  |  | (0.034) |
| is wall made of tin or better materials? |  |  |  |  | -0.163 |
|  |  |  |  |  | (0.273) |
| wall made of tin or tin mix in 1982 |  |  |  |  | 0.204 |
|  |  |  |  |  | (0.181) |
| is roof made of tin or better materials? |  |  |  |  | 0.342 |
|  |  |  |  |  | (0.185)+ |
| number of boats owned-82 |  |  |  |  | 0.013 |
|  |  |  |  |  | (0.126) |
| number of rooms in the HH-82 |  |  |  |  | 0.055 |
|  |  |  |  |  | (0.149) |
| ch_hhc_asset82 |  |  |  |  | 0.028 |
|  |  |  |  |  | (0.062) |
| number of cows owned-82 |  |  |  |  | -0.062 |
|  |  |  |  |  | (0.057) |
| use any fixed latrine? |  |  |  |  | 0.206 |
|  |  |  |  |  | (0.360) |
| HH uses tubewell water for drinking 1982 |  |  |  |  | 0.206 |
|  |  |  |  |  | (0.197) |
| HH uses river or ditch/canal water for drinking in 1982 |  |  |  |  | 0.186 |
|  |  |  |  |  | (0.213) |
| Head of HH's Years of Education, 82, Maktab=0 |  |  |  |  | 0.029 |
|  |  |  |  |  | (0.027) |
| Head of HH does Agriculture (=1 if Y, =0 if N) |  |  |  |  | -0.030 |
|  |  |  |  |  | (0.149) |
| Head of HH does Fishing (=1 if Y, =0 if N) |  |  |  |  | 0.269 |
|  |  |  |  |  | (0.296) |
| Head of HH's age in 1982 Census (=. if not head) |  |  |  |  | 0.005 |
|  |  |  |  |  | (0.005) |
| receive any remittance? |  |  |  |  | -0.040 |
|  |  |  |  |  | (0.171) |
| Constant | 22.395 | 20.084 | 18.785 | 18.880 | 17.976 |
|  | (0.192)*** | (2.033)*** | (2.040)*** | (2.029)*** | (2.252)*** |
|  |  |  |  |  |  |
| Observations | 4,296 | 4,296 | 4,296 | 4,296 | 3,792 |
| R-squared | 0.151 | 0.213 | 0.217 | 0.222 | 0.225 |
| F test statistics | 56.88 | 45.89 | 28.41 | 27.15 | 16.53 |
| Degrees of freedom | 9 | 16 | 27 | 29 | 46 |
| Robust standard errors in parentheses; *** p<0.001, ** p<0.01, * p<0.05, + p<0.1 | | | | |  |

| Full models, logistic regression of overweight/obese, Asian standard (BMI >= 23 kg/m2) | | | | | |
| --- | --- | --- | --- | --- | --- |
| VARIABLES | (1) | (2) | (3) | (4) | (5) |
|  |  |  |  |  |  |
| m2_mig_status2 = 0, Non-migrant | -1.568 | -1.433 | -1.423 | -1.396 | -1.380 |
|  | (0.135)*** | (0.147)*** | (0.147)*** | (0.148)*** | (0.155)*** |
| m2_mig_status2 = 1, Internal migrant | -0.721 | -0.705 | -0.687 | -0.640 | -0.591 |
|  | (0.136)*** | (0.147)*** | (0.148)*** | (0.151)*** | (0.159)*** |
| m2_mig_status2 = 3, Return int'l migrant | -0.228 | -0.207 | -0.203 | -0.212 | -0.192 |
|  | (0.225) | (0.248) | (0.247) | (0.243) | (0.254) |
| Age 25-29 | 1.008 | 1.089 | 1.056 | 1.048 | 1.044 |
|  | (0.169)*** | (0.172)*** | (0.172)*** | (0.171)*** | (0.179)*** |
| Age 30-34 | 1.220 | 1.236 | 1.181 | 1.151 | 1.142 |
|  | (0.170)*** | (0.174)*** | (0.177)*** | (0.177)*** | (0.190)*** |
| Age 35-39 | 1.295 | 1.320 | 1.245 | 1.200 | 1.158 |
|  | (0.178)*** | (0.186)*** | (0.190)*** | (0.192)*** | (0.209)*** |
| Age 40-44 | 1.179 | 1.359 | 1.253 | 1.203 | 1.148 |
|  | (0.208)*** | (0.217)*** | (0.220)*** | (0.221)*** | (0.227)*** |
| Age 45-49 | 0.952 | 1.305 | 1.204 | 1.156 | 1.043 |
|  | (0.216)*** | (0.220)*** | (0.227)*** | (0.230)*** | (0.247)*** |
| Age 50+ | 1.196 | 1.561 | 1.479 | 1.441 | 1.278 |
|  | (0.223)*** | (0.234)*** | (0.243)*** | (0.244)*** | (0.259)*** |
| Hindu religion |  | 0.191 | 0.212 | 0.225 | 0.271 |
|  |  | (0.165) | (0.167) | (0.167) | (0.196) |
| Schooling - 1-4 years |  | 0.414 | 0.390 | 0.386 | 0.313 |
|  |  | (0.204)* | (0.205)+ | (0.206)+ | (0.224) |
| Schooling - 5-9 years |  | 1.084 | 1.033 | 1.029 | 0.987 |
|  |  | (0.183)*** | (0.192)*** | (0.192)*** | (0.208)*** |
| Schooling - Lower Secondary |  | 1.562 | 1.495 | 1.490 | 1.394 |
|  |  | (0.213)*** | (0.229)*** | (0.228)*** | (0.247)*** |
| Schooling - Upper Secondarry |  | 1.411 | 1.326 | 1.308 | 1.178 |
|  |  | (0.221)*** | (0.241)*** | (0.240)*** | (0.259)*** |
| Schooling - College |  | 1.778 | 1.684 | 1.684 | 1.463 |
|  |  | (0.246)*** | (0.274)*** | (0.274)*** | (0.304)*** |
| Height (cm) |  | -0.002 | -0.003 | -0.003 | -0.004 |
|  |  | (0.010) | (0.010) | (0.010) | (0.010) |
| Household asssets, 1996 (logged) |  |  | 0.074 | 0.062 | 0.058 |
|  |  |  | (0.041)+ | (0.042) | (0.046) |
| Mother's schooling = 1, 1-4 years |  |  | -0.167 | -0.168 | -0.189 |
|  |  |  | (0.143) | (0.142) | (0.149) |
| Mother's schooling = 2, 5-9 years |  |  | 0.023 | 0.020 | 0.043 |
|  |  |  | (0.174) | (0.175) | (0.190) |
| Mother's schooling = 3, 10+ years |  |  | 0.315 | 0.329 | 0.229 |
|  |  |  | (0.407) | (0.415) | (0.446) |
| Father's schooling = 1, 1-4 years |  |  | 0.122 | 0.138 | 0.113 |
|  |  |  | (0.142) | (0.142) | (0.146) |
| Father's schooling = 2, 5-9 years |  |  | 0.031 | 0.035 | -0.030 |
|  |  |  | (0.142) | (0.141) | (0.156) |
| Father's schooling = 3, 10+ years |  |  | -0.143 | -0.131 | -0.187 |
|  |  |  | (0.201) | (0.202) | (0.242) |
| Younger brothers |  |  | 0.017 | -0.008 | -0.020 |
|  |  |  | (0.053) | (0.054) | (0.058) |
| Older brothers |  |  | 0.040 | 0.009 | 0.038 |
|  |  |  | (0.042) | (0.045) | (0.050) |
| Younger sisters |  |  | 0.060 | 0.063 | 0.120 |
|  |  |  | (0.051) | (0.051) | (0.055)* |
| Older sisters |  |  | 0.010 | 0.006 | -0.026 |
|  |  |  | (0.049) | (0.049) | (0.054) |
| Father ever international migrant |  |  |  | -0.058 | -0.013 |
|  |  |  |  | (0.197) | (0.213) |
| Any brother international migrant |  |  |  | 0.264 | 0.223 |
|  |  |  |  | (0.122)* | (0.127)+ |
| Number of households in a bari in 82 census |  |  |  |  | 0.019 |
|  |  |  |  |  | (0.011)+ |
| Family size-82 |  |  |  |  | -0.024 |
|  |  |  |  |  | (0.025) |
| is wall made of tin or better materials? |  |  |  |  | 0.216 |
|  |  |  |  |  | (0.198) |
| wall made of tin or tin mix in 1982 |  |  |  |  | 0.023 |
|  |  |  |  |  | (0.137) |
| is roof made of tin or better materials? |  |  |  |  | 0.281 |
|  |  |  |  |  | (0.170)+ |
| number of boats owned-82 |  |  |  |  | 0.061 |
|  |  |  |  |  | (0.102) |
| number of rooms in the HH-82 |  |  |  |  | 0.085 |
|  |  |  |  |  | (0.111) |
| ch_hhc_asset82 |  |  |  |  | 0.021 |
|  |  |  |  |  | (0.049) |
| number of cows owned-82 |  |  |  |  | -0.059 |
|  |  |  |  |  | (0.039) |
| use any fixed latrine? |  |  |  |  | 0.325 |
|  |  |  |  |  | (0.403) |
| HH uses tubewell water for drinking 1982 |  |  |  |  | 0.254 |
|  |  |  |  |  | (0.163) |
| HH uses river or ditch/canal water for drinking in 1982 |  |  |  |  | 0.114 |
|  |  |  |  |  | (0.181) |
| Head of HH's Years of Education, 82, Maktab=0 |  |  |  |  | -0.008 |
|  |  |  |  |  | (0.021) |
| Head of HH does Agriculture (=1 if Y, =0 if N) |  |  |  |  | -0.038 |
|  |  |  |  |  | (0.119) |
| Head of HH does Fishing (=1 if Y, =0 if N) |  |  |  |  | -0.274 |
|  |  |  |  |  | (0.266) |
| Head of HH's age in 1982 Census (=. if not head) |  |  |  |  | 0.006 |
|  |  |  |  |  | (0.004) |
| receive any remittance? |  |  |  |  | -0.087 |
|  |  |  |  |  | (0.136) |
| Constant | -0.715 | -1.573 | -2.249 | -2.184 | -2.931 |
|  | (0.168)*** | (1.637) | (1.644) | (1.647) | (1.831) |
|  |  |  |  |  |  |
| Observations | 4,296 | 4,296 | 4,296 | 4,296 | 3,792 |
| Pseudo R-squared | 0.0967 | 0.136 | 0.139 | 0.140 | 0.147 |
| Likelihood ratio chi-square | 241.6 | 333.5 | 351 | 358.3 | 337.2 |
| Degrees of freedom | 9 | 16 | 27 | 29 | 46 |
| Robust standard errors in parentheses, *** p<0.001, ** p<0.01, * p<0.05, + p<0.1 | | | | | |

| Full models, logistic regression of obese, Asian standard (BMI >= 27.5 kg/m2) | | | | | |
| --- | --- | --- | --- | --- | --- |
| VARIABLES | (1) | (2) | (3) | (4) | (5) |
|  |  |  |  |  |  |
| m2_mig_status2 = 0, Non-migrant | -0.797 | -0.786 | -0.790 | -0.765 | -0.796 |
|  | (0.268)** | (0.300)** | (0.299)** | (0.306)* | (0.315)* |
| m2_mig_status2 = 1, Internal migrant | -0.319 | -0.398 | -0.369 | -0.332 | -0.273 |
|  | (0.252) | (0.261) | (0.260) | (0.266) | (0.289) |
| m2_mig_status2 = 3, Return int'l migrant | -0.330 | -0.336 | -0.372 | -0.355 | -0.413 |
|  | (0.368) | (0.373) | (0.378) | (0.375) | (0.413) |
| Age 25-29 | 1.981 | 1.942 | 1.916 | 1.901 | 1.820 |
|  | (0.488)*** | (0.493)*** | (0.490)*** | (0.489)*** | (0.492)*** |
| Age 30-34 | 2.231 | 2.098 | 2.046 | 1.980 | 1.867 |
|  | (0.462)*** | (0.468)*** | (0.468)*** | (0.464)*** | (0.470)*** |
| Age 35-39 | 2.036 | 1.926 | 1.854 | 1.753 | 1.638 |
|  | (0.480)*** | (0.479)*** | (0.481)*** | (0.476)*** | (0.495)*** |
| Age 40-44 | 2.140 | 2.121 | 2.013 | 1.913 | 1.804 |
|  | (0.497)*** | (0.509)*** | (0.518)*** | (0.515)*** | (0.535)*** |
| Age 45-49 | 2.272 | 2.370 | 2.172 | 2.063 | 1.743 |
|  | (0.511)*** | (0.500)*** | (0.506)*** | (0.501)*** | (0.535)** |
| Age 50+ | 2.590 | 2.748 | 2.594 | 2.500 | 2.517 |
|  | (0.549)*** | (0.545)*** | (0.570)*** | (0.571)*** | (0.601)*** |
| Hindu religion |  | 0.247 | 0.258 | 0.279 | 0.184 |
|  |  | (0.288) | (0.285) | (0.284) | (0.326) |
| Schooling - 1-4 years |  | 0.505 | 0.448 | 0.434 | 0.335 |
|  |  | (0.472) | (0.472) | (0.476) | (0.536) |
| Schooling - 5-9 years |  | 1.052 | 0.883 | 0.876 | 0.871 |
|  |  | (0.394)** | (0.399)* | (0.400)* | (0.451)+ |
| Schooling - Lower Secondary |  | 1.582 | 1.330 | 1.329 | 1.167 |
|  |  | (0.432)*** | (0.452)** | (0.453)** | (0.512)* |
| Schooling - Upper Secondarry |  | 1.347 | 1.076 | 1.062 | 0.844 |
|  |  | (0.453)** | (0.471)* | (0.471)* | (0.560) |
| Schooling - College |  | 1.844 | 1.550 | 1.557 | 1.305 |
|  |  | (0.450)*** | (0.482)** | (0.486)** | (0.570)* |
| Height (cm) |  | -0.056 | -0.056 | -0.056 | -0.062 |
|  |  | (0.022)* | (0.020)** | (0.020)** | (0.022)** |
| Household asssets, 1996 (logged) |  |  | 0.129 | 0.115 | 0.080 |
|  |  |  | (0.090) | (0.092) | (0.098) |
| Mother's schooling = 1, 1-4 years |  |  | -0.111 | -0.115 | -0.233 |
|  |  |  | (0.267) | (0.267) | (0.264) |
| Mother's schooling = 2, 5-9 years |  |  | 0.045 | 0.047 | -0.065 |
|  |  |  | (0.280) | (0.278) | (0.289) |
| Mother's schooling = 3, 10+ years |  |  | -1.191 | -1.195 | -0.894 |
|  |  |  | (0.715)+ | (0.719)+ | (0.751) |
| Father's schooling = 1, 1-4 years |  |  | 0.062 | 0.078 | -0.105 |
|  |  |  | (0.260) | (0.260) | (0.278) |
| Father's schooling = 2, 5-9 years |  |  | 0.089 | 0.104 | 0.172 |
|  |  |  | (0.252) | (0.251) | (0.277) |
| Father's schooling = 3, 10+ years |  |  | 0.267 | 0.293 | 0.150 |
|  |  |  | (0.313) | (0.313) | (0.418) |
| Younger brothers |  |  | -0.028 | -0.066 | -0.069 |
|  |  |  | (0.088) | (0.093) | (0.112) |
| Older brothers |  |  | -0.093 | -0.144 | -0.132 |
|  |  |  | (0.085) | (0.099) | (0.111) |
| Younger sisters |  |  | 0.115 | 0.115 | 0.104 |
|  |  |  | (0.089) | (0.089) | (0.097) |
| Older sisters |  |  | 0.049 | 0.039 | 0.012 |
|  |  |  | (0.095) | (0.094) | (0.099) |
| Father ever international migrant |  |  |  | -0.404 | -0.075 |
|  |  |  |  | (0.445) | (0.455) |
| Any brother international migrant |  |  |  | 0.331 | 0.262 |
|  |  |  |  | (0.241) | (0.258) |
| Number of households in a bari in 82 census |  |  |  |  | -0.019 |
|  |  |  |  |  | (0.016) |
| Family size-82 |  |  |  |  | 0.089 |
|  |  |  |  |  | (0.048)+ |
| is wall made of tin or better materials? |  |  |  |  | -0.211 |
|  |  |  |  |  | (0.361) |
| wall made of tin or tin mix in 1982 |  |  |  |  | -0.105 |
|  |  |  |  |  | (0.256) |
| is roof made of tin or better materials? |  |  |  |  | 0.427 |
|  |  |  |  |  | (0.373) |
| number of boats owned-82 |  |  |  |  | 0.005 |
|  |  |  |  |  | (0.167) |
| number of rooms in the HH-82 |  |  |  |  | -0.424 |
|  |  |  |  |  | (0.259) |
| ch_hhc_asset82 |  |  |  |  | 0.034 |
|  |  |  |  |  | (0.105) |
| number of cows owned-82 |  |  |  |  | 0.009 |
|  |  |  |  |  | (0.084) |
| use any fixed latrine? |  |  |  |  | 0.574 |
|  |  |  |  |  | (0.830) |
| HH uses tubewell water for drinking 1982 |  |  |  |  | 0.535 |
|  |  |  |  |  | (0.341) |
| HH uses river or ditch/canal water for drinking in 1982 |  |  |  |  | 0.324 |
|  |  |  |  |  | (0.377) |
| Head of HH's Years of Education, 82, Maktab=0 |  |  |  |  | 0.033 |
|  |  |  |  |  | (0.040) |
| Head of HH does Agriculture (=1 if Y, =0 if N) |  |  |  |  | 0.002 |
|  |  |  |  |  | (0.218) |
| Head of HH does Fishing (=1 if Y, =0 if N) |  |  |  |  | 0.353 |
|  |  |  |  |  | (0.521) |
| Head of HH's age in 1982 Census (=. if not head) |  |  |  |  | 0.004 |
|  |  |  |  |  | (0.009) |
| receive any remittance? |  |  |  |  | -0.039 |
|  |  |  |  |  | (0.277) |
| Constant | -4.572 | 3.443 | 2.198 | 2.388 | 2.331 |
|  | (0.470)*** | (3.664) | (3.292) | (3.326) | (3.698) |
|  |  |  |  |  |  |
| Observations | 4,296 | 4,296 | 4,296 | 4,296 | 3,792 |
| Pseudo R-squared | 0.0431 | 0.0776 | 0.0858 | 0.0887 | 0.106 |
| Likelihood ratio chi-square | 40.74 | 86.95 | 97.07 | 103.1 | 157.5 |
| Degrees of freedom | 9 | 16 | 27 | 29 | 46 |
| Robust standard errors in parentheses, *** p<0.001, ** p<0.01, * p<0.05, + p<0.1 | | | | | |

| Full models, OLS regression of Average grip strength | | | | | |
| --- | --- | --- | --- | --- | --- |
|  | (1) | (2) | (3) | (4) | (5) |
|  |  |  |  |  |  |
| m2_mig_status2 = 0, Non-migrant | -3.928 | -3.194 | -3.181 | -3.139 | -3.156 |
|  | (0.668)*** | (0.578)*** | (0.582)*** | (0.583)*** | (0.576)*** |
| m2_mig_status2 = 1, Internal migrant | -2.286 | -2.109 | -2.200 | -2.138 | -2.281 |
|  | (0.672)*** | (0.591)*** | (0.593)*** | (0.602)*** | (0.608)*** |
| m2_mig_status2 = 3, Return int'l migrant | -1.268 | -1.660 | -1.699 | -1.692 | -1.392 |
|  | (0.887) | (0.814)* | (0.815)* | (0.813)* | (0.840)+ |
| Age 25-29 | -1.279 | -0.477 | -0.489 | -0.499 | -0.631 |
|  | (0.495)** | (0.458) | (0.462) | (0.460) | (0.471) |
| Age 30-34 | -1.422 | -0.684 | -0.650 | -0.676 | -0.523 |
|  | (0.473)** | (0.450) | (0.461) | (0.464) | (0.503) |
| Age 35-39 | -2.345 | -1.903 | -1.916 | -1.949 | -2.002 |
|  | (0.550)*** | (0.522)*** | (0.535)*** | (0.539)*** | (0.566)*** |
| Age 40-44 | -4.022 | -2.709 | -2.663 | -2.703 | -2.862 |
|  | (0.575)*** | (0.543)*** | (0.574)*** | (0.582)*** | (0.606)*** |
| Age 45-49 | -5.950 | -4.072 | -4.006 | -4.044 | -4.180 |
|  | (0.677)*** | (0.624)*** | (0.621)*** | (0.626)*** | (0.658)*** |
| Age 50+ | -8.173 | -6.591 | -6.421 | -6.454 | -6.591 |
|  | (0.606)*** | (0.577)*** | (0.616)*** | (0.621)*** | (0.642)*** |
| Hindu religion |  | -0.492 | -0.432 | -0.422 | -0.856 |
|  |  | (0.415) | (0.424) | (0.426) | (0.501)+ |
| Schooling - 1-4 years |  | 1.354 | 1.398 | 1.396 | 1.333 |
|  |  | (0.494)** | (0.491)** | (0.491)** | (0.507)** |
| Schooling - 5-9 years |  | 2.067 | 2.255 | 2.246 | 2.288 |
|  |  | (0.451)*** | (0.464)*** | (0.465)*** | (0.484)*** |
| Schooling - Lower Secondary |  | 2.678 | 2.851 | 2.833 | 2.494 |
|  |  | (0.569)*** | (0.592)*** | (0.595)*** | (0.629)*** |
| Schooling - Upper Secondarry |  | 2.962 | 3.151 | 3.120 | 2.831 |
|  |  | (0.707)*** | (0.742)*** | (0.741)*** | (0.772)*** |
| Schooling - College |  | 2.683 | 2.802 | 2.790 | 2.385 |
|  |  | (0.646)*** | (0.688)*** | (0.687)*** | (0.747)** |
| Height (cm) |  | 0.359 | 0.363 | 0.363 | 0.371 |
|  |  | (0.026)*** | (0.025)*** | (0.025)*** | (0.027)*** |
| Household asssets, 1996 (logged) |  |  | -0.128 | -0.137 | -0.159 |
|  |  |  | (0.112) | (0.113) | (0.124) |
| Mother's schooling = 1, 1-4 years |  |  | -0.216 | -0.219 | -0.038 |
|  |  |  | (0.426) | (0.427) | (0.445) |
| Mother's schooling = 2, 5-9 years |  |  | 1.220 | 1.218 | 1.076 |
|  |  |  | (0.545)* | (0.546)* | (0.598)+ |
| Mother's schooling = 3, 10+ years |  |  | 2.079 | 2.088 | 1.758 |
|  |  |  | (1.382) | (1.392) | (1.491) |
| Father's schooling = 1, 1-4 years |  |  | -0.055 | -0.038 | 0.113 |
|  |  |  | (0.394) | (0.395) | (0.411) |
| Father's schooling = 2, 5-9 years |  |  | 0.167 | 0.167 | 0.018 |
|  |  |  | (0.450) | (0.451) | (0.460) |
| Father's schooling = 3, 10+ years |  |  | -1.126 | -1.118 | -1.032 |
|  |  |  | (0.594)+ | (0.595)+ | (0.662) |
| Younger brothers |  |  | 0.324 | 0.299 | 0.109 |
|  |  |  | (0.159)* | (0.160)+ | (0.171) |
| Older brothers |  |  | -0.043 | -0.071 | -0.196 |
|  |  |  | (0.133) | (0.141) | (0.158) |
| Younger sisters |  |  | -0.050 | -0.048 | -0.072 |
|  |  |  | (0.150) | (0.150) | (0.156) |
| Older sisters |  |  | 0.216 | 0.210 | 0.079 |
|  |  |  | (0.132) | (0.132) | (0.146) |
| Father ever international migrant |  |  |  | -0.016 | -0.577 |
|  |  |  |  | (0.644) | (0.696) |
| Any brother international migrant |  |  |  | 0.245 | 0.321 |
|  |  |  |  | (0.361) | (0.375) |
| Number of households in a bari in 82 census |  |  |  |  | -0.015 |
|  |  |  |  |  | (0.027) |
| Family size-82 |  |  |  |  | 0.118 |
|  |  |  |  |  | (0.072)+ |
| is wall made of tin or better materials? |  |  |  |  | 0.274 |
|  |  |  |  |  | (0.633) |
| wall made of tin or tin mix in 1982 |  |  |  |  | 0.888 |
|  |  |  |  |  | (0.419)* |
| is roof made of tin or better materials? |  |  |  |  | -0.011 |
|  |  |  |  |  | (0.439) |
| number of boats owned-82 |  |  |  |  | -0.349 |
|  |  |  |  |  | (0.275) |
| number of rooms in the HH-82 |  |  |  |  | 0.010 |
|  |  |  |  |  | (0.351) |
| ch_hhc_asset82 |  |  |  |  | 0.007 |
|  |  |  |  |  | (0.137) |
| number of cows owned-82 |  |  |  |  | 0.157 |
|  |  |  |  |  | (0.111) |
| use any fixed latrine? |  |  |  |  | -1.510 |
|  |  |  |  |  | (0.846)+ |
| HH uses tubewell water for drinking 1982 |  |  |  |  | 0.355 |
|  |  |  |  |  | (0.502) |
| HH uses river or ditch/canal water for drinking in 1982 |  |  |  |  | 0.593 |
|  |  |  |  |  | (0.533) |
| Head of HH's Years of Education, 82, Maktab=0 |  |  |  |  | -0.010 |
|  |  |  |  |  | (0.060) |
| Head of HH does Agriculture (=1 if Y, =0 if N) |  |  |  |  | -0.445 |
|  |  |  |  |  | (0.353) |
| Head of HH does Fishing (=1 if Y, =0 if N) |  |  |  |  | 0.968 |
|  |  |  |  |  | (0.661) |
| Head of HH's age in 1982 Census (=. if not head) |  |  |  |  | 0.006 |
|  |  |  |  |  | (0.012) |
| receive any remittance? |  |  |  |  | 0.089 |
|  |  |  |  |  | (0.391) |
| Constant | 44.841 | -16.654 | -16.383 | -16.368 | -16.744 |
|  | (0.684)*** | (4.245)*** | (4.361)*** | (4.360)*** | (4.771)*** |
|  |  |  |  |  |  |
| Observations | 3,754 | 3,754 | 3,754 | 3,754 | 3,300 |
| R-squared | 0.163 | 0.286 | 0.292 | 0.292 | 0.308 |
| F test statistics | 41.69 | 45.68 | 28.94 | 27.01 | 18.57 |
| Degrees of freedom | 9 | 16 | 27 | 29 | 46 |
| Robust standard errors in parentheses; *** p<0.001, ** p<0.01, * p<0.05, + p<0.1 | | | | |  |

| Full models, logistic regression of hypertension stage 1 or worse | | | | |
| --- | --- | --- | --- | --- |
|  | (1) | (2) | (3) | (4) |
| m2_mig_status2 = 0, Non-migrant | -0.775 | -0.631 | -0.636 | -0.728 |
|  | (0.296)** | (0.299)* | (0.297)* | (0.297)* |
| m2_mig_status2 = 1, Internal migrant | 0.026 | 0.107 | 0.078 | -0.037 |
|  | (0.298) | (0.307) | (0.303) | (0.303) |
| m2_mig_status2 = 3, Return int'l migrant | -0.373 | -0.426 | -0.454 | -0.522 |
|  | (0.409) | (0.406) | (0.403) | (0.405) |
| Age 25-29 | 0.247 | 0.353 | 0.315 | 0.307 |
|  | (0.303) | (0.312) | (0.316) | (0.316) |
| Age 30-34 | 1.047 | 1.096 | 1.056 | 1.053 |
|  | (0.284)*** | (0.296)*** | (0.300)*** | (0.302)*** |
| Age 35-39 | 0.992 | 1.045 | 0.969 | 0.963 |
|  | (0.306)** | (0.317)*** | (0.333)** | (0.336)** |
| Age 40-44 | 1.326 | 1.548 | 1.488 | 1.480 |
|  | (0.360)*** | (0.374)*** | (0.392)*** | (0.396)*** |
| Age 45-49 | 1.231 | 1.563 | 1.506 | 1.490 |
|  | (0.334)*** | (0.348)*** | (0.368)*** | (0.371)*** |
| Age 50+ = o, |  |  |  |  |
|  |  |  |  |  |
| Hindu religion |  | 0.179 | 0.168 | 0.139 |
|  |  | (0.220) | (0.219) | (0.217) |
| Schooling - 1-4 years |  | 0.595 | 0.557 | 0.547 |
|  |  | (0.328)+ | (0.327)+ | (0.326)+ |
| Schooling - 5-9 years |  | 0.825 | 0.753 | 0.757 |
|  |  | (0.294)** | (0.301)* | (0.299)* |
| Schooling - Lower Secondary |  | 1.465 | 1.335 | 1.366 |
|  |  | (0.319)*** | (0.333)*** | (0.331)*** |
| Schooling - Upper Secondarry |  | 1.034 | 0.903 | 0.947 |
|  |  | (0.365)** | (0.387)* | (0.383)* |
| Schooling - College |  | 0.777 | 0.634 | 0.644 |
|  |  | (0.354)* | (0.386) | (0.385)+ |
| Height (cm) |  | 0.026 | 0.023 | 0.023 |
|  |  | (0.014)+ | (0.014)+ | (0.014)+ |
| Household asssets, 1996 (logged) |  |  | 0.002 | 0.015 |
|  |  |  | (0.064) | (0.065) |
| Mother's schooling = 1, 1-4 years |  |  | -0.302 | -0.310 |
|  |  |  | (0.224) | (0.226) |
| Mother's schooling = 2, 5-9 years |  |  | 0.241 | 0.279 |
|  |  |  | (0.249) | (0.249) |
| Mother's schooling = 3, 10+ years |  |  | 0.074 | 0.099 |
|  |  |  | (0.620) | (0.621) |
| Father's schooling = 1, 1-4 years |  |  | 0.421 | 0.415 |
|  |  |  | (0.228)+ | (0.228)+ |
| Father's schooling = 2, 5-9 years |  |  | 0.375 | 0.392 |
|  |  |  | (0.211)+ | (0.213)+ |
| Father's schooling = 3, 10+ years |  |  | 0.096 | 0.091 |
|  |  |  | (0.295) | (0.294) |
| Younger brothers |  |  | 0.036 | 0.063 |
|  |  |  | (0.073) | (0.075) |
| Older brothers |  |  | 0.084 | 0.101 |
|  |  |  | (0.069) | (0.071) |
| Younger sisters |  |  | 0.033 | 0.029 |
|  |  |  | (0.082) | (0.083) |
| Older sisters |  |  | -0.050 | -0.048 |
|  |  |  | (0.089) | (0.088) |
| Father ever international migrant |  |  |  | -0.545 |
|  |  |  |  | (0.391) |
| Any brother international migrant |  |  |  | -0.275 |
|  |  |  |  | (0.193) |
| Age 50+ | 1.617 | 1.955 | 1.941 | 1.928 |
|  | (0.339)*** | (0.357)*** | (0.374)*** | (0.372)*** |
| Constant | -2.776 | -8.022 | -7.779 | -7.696 |
|  | (0.340)*** | (2.341)*** | (2.320)*** | (2.301)*** |
|  |  |  |  |  |
| Observations | 3,760 | 3,760 | 3,760 | 3,760 |
| Pseudo R-squared | 0.0420 | 0.0668 | 0.0749 | 0.0779 |
| Likelihood ratio chi-square | 45.78 | 79.28 | 92.63 | 99.32 |
| Degrees of freedom | 9 | 16 | 27 | 29 |
| Robust standard errors in parentheses, *** p<0.001, ** p<0.01, * p<0.05, + p<0.1 | | | | |

| Full models, OLS regression of standardized score on 12-item CES-D, all items | | | | | |
| --- | --- | --- | --- | --- | --- |
| VARIABLES | (1) | (2) | (3) | (4) | (5) |
|  |  |  |  |  |  |
| m2_mig_status2 = 0, Non-migrant | -0.029 | -0.111 | -0.125 | -0.121 | -0.119 |
|  | (0.068) | (0.072) | (0.071)+ | (0.071)+ | (0.075) |
| m2_mig_status2 = 1, Internal migrant | -0.209 | -0.253 | -0.256 | -0.248 | -0.248 |
|  | (0.075)** | (0.075)*** | (0.075)*** | (0.075)*** | (0.079)** |
| m2_mig_status2 = 3, Return int'l migrant | -0.141 | -0.162 | -0.159 | -0.162 | -0.161 |
|  | (0.115) | (0.109) | (0.108) | (0.107) | (0.112) |
| Age 25-29 | 0.091 | 0.068 | 0.076 | 0.071 | 0.078 |
|  | (0.065) | (0.066) | (0.066) | (0.066) | (0.067) |
| Age 30-34 | 0.103 | 0.104 | 0.112 | 0.100 | 0.124 |
|  | (0.070) | (0.072) | (0.071) | (0.071) | (0.076) |
| Age 35-39 | 0.135 | 0.135 | 0.148 | 0.131 | 0.123 |
|  | (0.079)+ | (0.082)+ | (0.083)+ | (0.081) | (0.084) |
| Age 40-44 | 0.283 | 0.232 | 0.233 | 0.214 | 0.202 |
|  | (0.076)*** | (0.078)** | (0.082)** | (0.082)** | (0.087)* |
| Age 45-49 | 0.308 | 0.213 | 0.210 | 0.191 | 0.179 |
|  | (0.083)*** | (0.084)* | (0.088)* | (0.089)* | (0.094)+ |
| Age 50+ | 0.602 | 0.519 | 0.498 | 0.481 | 0.459 |
|  | (0.096)*** | (0.099)*** | (0.104)*** | (0.104)*** | (0.109)*** |
| Hindu religion |  | -0.072 | -0.082 | -0.080 | -0.112 |
|  |  | (0.058) | (0.058) | (0.058) | (0.067)+ |
| Schooling - 1-4 years |  | 0.051 | 0.052 | 0.050 | 0.002 |
|  |  | (0.070) | (0.070) | (0.070) | (0.072) |
| Schooling - 5-9 years |  | -0.232 | -0.224 | -0.228 | -0.225 |
|  |  | (0.065)*** | (0.067)*** | (0.067)*** | (0.070)** |
| Schooling - Lower Secondary |  | -0.413 | -0.399 | -0.401 | -0.440 |
|  |  | (0.086)*** | (0.092)*** | (0.092)*** | (0.096)*** |
| Schooling - Upper Secondarry |  | -0.237 | -0.209 | -0.213 | -0.230 |
|  |  | (0.099)* | (0.110)+ | (0.110)+ | (0.113)* |
| Schooling - College |  | -0.383 | -0.363 | -0.366 | -0.379 |
|  |  | (0.098)*** | (0.108)*** | (0.107)*** | (0.113)*** |
| Height (cm) |  | -0.002 | -0.002 | -0.002 | -0.003 |
|  |  | (0.004) | (0.004) | (0.004) | (0.004) |
| Household asssets, 1996 (logged) |  |  | -0.008 | -0.010 | -0.009 |
|  |  |  | (0.016) | (0.017) | (0.018) |
| Mother's schooling = 1, 1-4 years |  |  | 0.036 | 0.036 | 0.018 |
|  |  |  | (0.063) | (0.063) | (0.067) |
| Mother's schooling = 2, 5-9 years |  |  | -0.138 | -0.134 | -0.188 |
|  |  |  | (0.078)+ | (0.078)+ | (0.080)* |
| Mother's schooling = 3, 10+ years |  |  | 0.179 | 0.188 | 0.263 |
|  |  |  | (0.265) | (0.262) | (0.273) |
| Father's schooling = 1, 1-4 years |  |  | -0.032 | -0.026 | -0.017 |
|  |  |  | (0.057) | (0.057) | (0.060) |
| Father's schooling = 2, 5-9 years |  |  | 0.019 | 0.021 | 0.057 |
|  |  |  | (0.062) | (0.061) | (0.066) |
| Father's schooling = 3, 10+ years |  |  | 0.015 | 0.017 | 0.070 |
|  |  |  | (0.086) | (0.086) | (0.094) |
| Younger brothers |  |  | -0.015 | -0.022 | -0.018 |
|  |  |  | (0.019) | (0.020) | (0.023) |
| Older brothers |  |  | -0.020 | -0.029 | -0.024 |
|  |  |  | (0.017) | (0.018) | (0.020) |
| Younger sisters |  |  | 0.005 | 0.006 | 0.015 |
|  |  |  | (0.021) | (0.021) | (0.023) |
| Older sisters |  |  | -0.025 | -0.027 | -0.026 |
|  |  |  | (0.020) | (0.020) | (0.022) |
| Father ever international migrant |  |  |  | -0.085 | -0.087 |
|  |  |  |  | (0.093) | (0.100) |
| Any brother international migrant |  |  |  | 0.063 | 0.051 |
|  |  |  |  | (0.052) | (0.054) |
| Number of households in a bari in 82 census |  |  |  |  | 0.009 |
|  |  |  |  |  | (0.004)* |
| Family size-82 |  |  |  |  | -0.003 |
|  |  |  |  |  | (0.010) |
| is wall made of tin or better materials? |  |  |  |  | -0.012 |
|  |  |  |  |  | (0.090) |
| wall made of tin or tin mix in 1982 |  |  |  |  | -0.035 |
|  |  |  |  |  | (0.062) |
| is roof made of tin or better materials? |  |  |  |  | 0.052 |
|  |  |  |  |  | (0.064) |
| number of boats owned-82 |  |  |  |  | -0.002 |
|  |  |  |  |  | (0.039) |
| number of rooms in the HH-82 |  |  |  |  | 0.013 |
|  |  |  |  |  | (0.048) |
| ch_hhc_asset82 |  |  |  |  | 0.008 |
|  |  |  |  |  | (0.020) |
| number of cows owned-82 |  |  |  |  | 0.025 |
|  |  |  |  |  | (0.018) |
| use any fixed latrine? |  |  |  |  | 0.228 |
|  |  |  |  |  | (0.106)* |
| HH uses tubewell water for drinking 1982 |  |  |  |  | -0.049 |
|  |  |  |  |  | (0.074) |
| HH uses river or ditch/canal water for drinking in 1982 |  |  |  |  | -0.165 |
|  |  |  |  |  | (0.078)* |
| Head of HH's Years of Education, 82, Maktab=0 |  |  |  |  | -0.010 |
|  |  |  |  |  | (0.009) |
| Head of HH does Agriculture (=1 if Y, =0 if N) |  |  |  |  | 0.036 |
|  |  |  |  |  | (0.053) |
| Head of HH does Fishing (=1 if Y, =0 if N) |  |  |  |  | 0.234 |
|  |  |  |  |  | (0.090)** |
| Head of HH's age in 1982 Census (=. if not head) |  |  |  |  | 0.001 |
|  |  |  |  |  | (0.002) |
| receive any remittance? |  |  |  |  | -0.062 |
|  |  |  |  |  | (0.058) |
| Constant | -0.011 | 0.625 | 0.780 | 0.790 | 0.571 |
|  | (0.079) | (0.612) | (0.620) | (0.619) | (0.651) |
|  |  |  |  |  |  |
| Observations | 4,017 | 4,017 | 4,017 | 4,017 | 3,550 |
| R-squared | 0.042 | 0.067 | 0.072 | 0.073 | 0.088 |
| F test statistics | 9.325 | 9.218 | 6.146 | 5.908 | 4.291 |
| Degrees of freedom | 9 | 16 | 27 | 29 | 46 |
| Robust standard errors in parentheses, *** p<0.001, ** p<0.01, * p<0.05, + p<0.1 | | | | | |

| Full models, OLS regression of standardized score on 12-item CES-D, positive items | | | | | |
| --- | --- | --- | --- | --- | --- |
| VARIABLES | (1) | (2) | (3) | (4) | (5) |
|  |  |  |  |  |  |
| m2_mig_status2 = 0, Non-migrant | 0.115 | 0.013 | 0.013 | 0.014 | 0.008 |
|  | (0.069)+ | (0.071) | (0.070) | (0.070) | (0.075) |
| m2_mig_status2 = 1, Internal migrant | -0.086 | -0.133 | -0.127 | -0.125 | -0.131 |
|  | (0.073) | (0.074)+ | (0.074)+ | (0.074)+ | (0.080)+ |
| m2_mig_status2 = 3, Return int'l migrant | 0.022 | 0.006 | 0.009 | 0.008 | 0.000 |
|  | (0.118) | (0.119) | (0.120) | (0.120) | (0.130) |
| Age 25-29 | 0.148 | 0.114 | 0.112 | 0.108 | 0.130 |
|  | (0.066)* | (0.066)+ | (0.066)+ | (0.066) | (0.068)+ |
| Age 30-34 | 0.160 | 0.154 | 0.152 | 0.144 | 0.181 |
|  | (0.069)* | (0.069)* | (0.070)* | (0.070)* | (0.074)* |
| Age 35-39 | 0.229 | 0.224 | 0.222 | 0.212 | 0.214 |
|  | (0.075)** | (0.076)** | (0.079)** | (0.079)** | (0.082)** |
| Age 40-44 | 0.329 | 0.263 | 0.239 | 0.227 | 0.241 |
|  | (0.081)*** | (0.081)** | (0.086)** | (0.086)** | (0.089)** |
| Age 45-49 | 0.338 | 0.215 | 0.185 | 0.172 | 0.176 |
|  | (0.091)*** | (0.091)* | (0.097)+ | (0.097)+ | (0.102)+ |
| Age 50+ | 0.522 | 0.409 | 0.359 | 0.347 | 0.350 |
|  | (0.090)*** | (0.090)*** | (0.096)*** | (0.096)*** | (0.102)*** |
| Hindu religion |  | 0.029 | 0.033 | 0.032 | -0.045 |
|  |  | (0.062) | (0.063) | (0.063) | (0.073) |
| Schooling - 1-4 years |  | 0.035 | 0.032 | 0.031 | -0.028 |
|  |  | (0.074) | (0.074) | (0.074) | (0.077) |
| Schooling - 5-9 years |  | -0.259 | -0.269 | -0.271 | -0.274 |
|  |  | (0.070)*** | (0.073)*** | (0.073)*** | (0.076)*** |
| Schooling - Lower Secondary |  | -0.494 | -0.513 | -0.513 | -0.583 |
|  |  | (0.089)*** | (0.096)*** | (0.096)*** | (0.098)*** |
| Schooling - Upper Secondarry |  | -0.354 | -0.357 | -0.357 | -0.381 |
|  |  | (0.095)*** | (0.103)*** | (0.103)*** | (0.108)*** |
| Schooling - College |  | -0.459 | -0.466 | -0.468 | -0.533 |
|  |  | (0.097)*** | (0.105)*** | (0.105)*** | (0.112)*** |
| Height (cm) |  | -0.003 | -0.004 | -0.003 | -0.003 |
|  |  | (0.004) | (0.004) | (0.004) | (0.004) |
| Household asssets, 1996 (logged) |  |  | 0.020 | 0.020 | 0.015 |
|  |  |  | (0.017) | (0.017) | (0.019) |
| Mother's schooling = 1, 1-4 years |  |  | 0.024 | 0.023 | -0.023 |
|  |  |  | (0.062) | (0.062) | (0.066) |
| Mother's schooling = 2, 5-9 years |  |  | -0.056 | -0.051 | -0.102 |
|  |  |  | (0.080) | (0.081) | (0.084) |
| Mother's schooling = 3, 10+ years |  |  | 0.212 | 0.217 | 0.245 |
|  |  |  | (0.245) | (0.243) | (0.259) |
| Father's schooling = 1, 1-4 years |  |  | -0.007 | -0.004 | 0.014 |
|  |  |  | (0.059) | (0.059) | (0.061) |
| Father's schooling = 2, 5-9 years |  |  | -0.011 | -0.010 | 0.017 |
|  |  |  | (0.061) | (0.061) | (0.065) |
| Father's schooling = 3, 10+ years |  |  | -0.102 | -0.101 | -0.040 |
|  |  |  | (0.082) | (0.082) | (0.093) |
| Younger brothers |  |  | -0.006 | -0.008 | -0.007 |
|  |  |  | (0.020) | (0.021) | (0.023) |
| Older brothers |  |  | -0.006 | -0.010 | -0.008 |
|  |  |  | (0.017) | (0.018) | (0.020) |
| Younger sisters |  |  | 0.002 | 0.002 | 0.004 |
|  |  |  | (0.021) | (0.021) | (0.022) |
| Older sisters |  |  | -0.045 | -0.047 | -0.045 |
|  |  |  | (0.020)* | (0.020)* | (0.022)* |
| Father ever international migrant |  |  |  | -0.079 | -0.092 |
|  |  |  |  | (0.095) | (0.102) |
| Any brother international migrant |  |  |  | 0.021 | 0.009 |
|  |  |  |  | (0.052) | (0.054) |
| Number of households in a bari in 82 census |  |  |  |  | 0.004 |
|  |  |  |  |  | (0.004) |
| Family size-82 |  |  |  |  | -0.003 |
|  |  |  |  |  | (0.010) |
| is wall made of tin or better materials? |  |  |  |  | 0.039 |
|  |  |  |  |  | (0.093) |
| wall made of tin or tin mix in 1982 |  |  |  |  | -0.026 |
|  |  |  |  |  | (0.065) |
| is roof made of tin or better materials? |  |  |  |  | 0.078 |
|  |  |  |  |  | (0.060) |
| number of boats owned-82 |  |  |  |  | -0.007 |
|  |  |  |  |  | (0.042) |
| number of rooms in the HH-82 |  |  |  |  | -0.009 |
|  |  |  |  |  | (0.050) |
| ch_hhc_asset82 |  |  |  |  | 0.007 |
|  |  |  |  |  | (0.021) |
| number of cows owned-82 |  |  |  |  | 0.028 |
|  |  |  |  |  | (0.018) |
| use any fixed latrine? |  |  |  |  | 0.143 |
|  |  |  |  |  | (0.124) |
| HH uses tubewell water for drinking 1982 |  |  |  |  | -0.019 |
|  |  |  |  |  | (0.076) |
| HH uses river or ditch/canal water for drinking in 1982 |  |  |  |  | -0.074 |
|  |  |  |  |  | (0.082) |
| Head of HH's Years of Education, 82, Maktab=0 |  |  |  |  | -0.004 |
|  |  |  |  |  | (0.009) |
| Head of HH does Agriculture (=1 if Y, =0 if N) |  |  |  |  | 0.043 |
|  |  |  |  |  | (0.050) |
| Head of HH does Fishing (=1 if Y, =0 if N) |  |  |  |  | 0.240 |
|  |  |  |  |  | (0.096)* |
| Head of HH's age in 1982 Census (=. if not head) |  |  |  |  | 0.001 |
|  |  |  |  |  | (0.002) |
| receive any remittance? |  |  |  |  | -0.042 |
|  |  |  |  |  | (0.060) |
| Constant | -0.174 | 0.642 | 0.564 | 0.567 | 0.315 |
|  | (0.078)* | (0.616) | (0.630) | (0.631) | (0.675) |
|  |  |  |  |  |  |
| Observations | 4,019 | 4,019 | 4,019 | 4,019 | 3,552 |
| R-squared | 0.035 | 0.069 | 0.073 | 0.073 | 0.083 |
| F test statistics | 8.509 | 10.11 | 6.757 | 6.344 | 4.151 |
| Degrees of freedom | 9 | 16 | 27 | 29 | 46 |
| Robust standard errors in parentheses, *** p<0.001, ** p<0.01, * p<0.05, + p<0.1 | | | | | |

| Full models, OLS regression of standardized score on 12-item CES-D, negative items | | | | | |
| --- | --- | --- | --- | --- | --- |
| VARIABLES | (1) | (2) | (3) | (4) | (5) |
|  |  |  |  |  |  |
| m2_mig_status2 = 0, Non-migrant | -0.145 | -0.182 | -0.204 | -0.198 | -0.191 |
|  | (0.065)* | (0.069)** | (0.069)** | (0.069)** | (0.072)** |
| m2_mig_status2 = 1, Internal migrant | -0.248 | -0.273 | -0.284 | -0.272 | -0.267 |
|  | (0.070)*** | (0.071)*** | (0.071)*** | (0.071)*** | (0.074)*** |
| m2_mig_status2 = 3, Return int'l migrant | -0.237 | -0.255 | -0.252 | -0.257 | -0.249 |
|  | (0.137)+ | (0.132)+ | (0.131)+ | (0.129)* | (0.133)+ |
| Age 25-29 | 0.012 | 0.006 | 0.019 | 0.015 | 0.006 |
|  | (0.063) | (0.065) | (0.065) | (0.065) | (0.068) |
| Age 30-34 | 0.019 | 0.026 | 0.039 | 0.028 | 0.033 |
|  | (0.067) | (0.069) | (0.069) | (0.069) | (0.074) |
| Age 35-39 | 0.008 | 0.012 | 0.033 | 0.017 | 0.003 |
|  | (0.084) | (0.086) | (0.088) | (0.086) | (0.091) |
| Age 40-44 | 0.149 | 0.128 | 0.150 | 0.131 | 0.100 |
|  | (0.077)+ | (0.080) | (0.083)+ | (0.083) | (0.089) |
| Age 45-49 | 0.181 | 0.140 | 0.163 | 0.144 | 0.121 |
|  | (0.078)* | (0.082)+ | (0.085)+ | (0.085)+ | (0.090) |
| Age 50+ | 0.473 | 0.442 | 0.454 | 0.437 | 0.401 |
|  | (0.110)*** | (0.115)*** | (0.118)*** | (0.117)*** | (0.122)*** |
| Hindu religion |  | -0.137 | -0.155 | -0.152 | -0.134 |
|  |  | (0.061)* | (0.061)* | (0.061)* | (0.068)* |
| Schooling - 1-4 years |  | 0.048 | 0.052 | 0.050 | 0.027 |
|  |  | (0.080) | (0.080) | (0.081) | (0.082) |
| Schooling - 5-9 years |  | -0.131 | -0.110 | -0.114 | -0.107 |
|  |  | (0.074)+ | (0.078) | (0.078) | (0.081) |
| Schooling - Lower Secondary |  | -0.205 | -0.167 | -0.170 | -0.169 |
|  |  | (0.098)* | (0.106) | (0.106) | (0.112) |
| Schooling - Upper Secondarry |  | -0.057 | -0.010 | -0.017 | -0.022 |
|  |  | (0.111) | (0.123) | (0.124) | (0.127) |
| Schooling - College |  | -0.190 | -0.153 | -0.156 | -0.119 |
|  |  | (0.102)+ | (0.113) | (0.112) | (0.117) |
| Height (cm) |  | -0.001 | -0.000 | -0.000 | -0.002 |
|  |  | (0.004) | (0.004) | (0.004) | (0.004) |
| Household asssets, 1996 (logged) |  |  | -0.030 | -0.033 | -0.027 |
|  |  |  | (0.017)+ | (0.017)+ | (0.019) |
| Mother's schooling = 1, 1-4 years |  |  | 0.035 | 0.035 | 0.049 |
|  |  |  | (0.071) | (0.071) | (0.076) |
| Mother's schooling = 2, 5-9 years |  |  | -0.164 | -0.161 | -0.201 |
|  |  |  | (0.071)* | (0.071)* | (0.073)** |
| Mother's schooling = 3, 10+ years |  |  | 0.090 | 0.099 | 0.190 |
|  |  |  | (0.216) | (0.213) | (0.220) |
| Father's schooling = 1, 1-4 years |  |  | -0.043 | -0.036 | -0.038 |
|  |  |  | (0.055) | (0.055) | (0.060) |
| Father's schooling = 2, 5-9 years |  |  | 0.039 | 0.041 | 0.074 |
|  |  |  | (0.062) | (0.062) | (0.067) |
| Father's schooling = 3, 10+ years |  |  | 0.113 | 0.116 | 0.145 |
|  |  |  | (0.088) | (0.088) | (0.096) |
| Younger brothers |  |  | -0.018 | -0.026 | -0.021 |
|  |  |  | (0.020) | (0.021) | (0.022) |
| Older brothers |  |  | -0.025 | -0.036 | -0.030 |
|  |  |  | (0.018) | (0.019)+ | (0.021) |
| Younger sisters |  |  | 0.006 | 0.007 | 0.020 |
|  |  |  | (0.023) | (0.023) | (0.024) |
| Older sisters |  |  | 0.002 | -0.000 | -0.001 |
|  |  |  | (0.020) | (0.020) | (0.021) |
| Father ever international migrant |  |  |  | -0.062 | -0.055 |
|  |  |  |  | (0.086) | (0.092) |
| Any brother international migrant |  |  |  | 0.079 | 0.070 |
|  |  |  |  | (0.055) | (0.055) |
| Number of households in a bari in 82 census |  |  |  |  | 0.011 |
|  |  |  |  |  | (0.005)* |
| Family size-82 |  |  |  |  | -0.002 |
|  |  |  |  |  | (0.010) |
| is wall made of tin or better materials? |  |  |  |  | -0.053 |
|  |  |  |  |  | (0.084) |
| wall made of tin or tin mix in 1982 |  |  |  |  | -0.031 |
|  |  |  |  |  | (0.064) |
| is roof made of tin or better materials? |  |  |  |  | 0.012 |
|  |  |  |  |  | (0.069) |
| number of boats owned-82 |  |  |  |  | 0.003 |
|  |  |  |  |  | (0.039) |
| number of rooms in the HH-82 |  |  |  |  | 0.028 |
|  |  |  |  |  | (0.050) |
| ch_hhc_asset82 |  |  |  |  | 0.006 |
|  |  |  |  |  | (0.020) |
| number of cows owned-82 |  |  |  |  | 0.013 |
|  |  |  |  |  | (0.017) |
| use any fixed latrine? |  |  |  |  | 0.226 |
|  |  |  |  |  | (0.129)+ |
| HH uses tubewell water for drinking 1982 |  |  |  |  | -0.058 |
|  |  |  |  |  | (0.080) |
| HH uses river or ditch/canal water for drinking in 1982 |  |  |  |  | -0.190 |
|  |  |  |  |  | (0.084)* |
| Head of HH's Years of Education, 82, Maktab=0 |  |  |  |  | -0.012 |
|  |  |  |  |  | (0.009) |
| Head of HH does Agriculture (=1 if Y, =0 if N) |  |  |  |  | 0.017 |
|  |  |  |  |  | (0.053) |
| Head of HH does Fishing (=1 if Y, =0 if N) |  |  |  |  | 0.150 |
|  |  |  |  |  | (0.094) |
| Head of HH's age in 1982 Census (=. if not head) |  |  |  |  | 0.001 |
|  |  |  |  |  | (0.002) |
| receive any remittance? |  |  |  |  | -0.059 |
|  |  |  |  |  | (0.055) |
| Constant | 0.135 | 0.404 | 0.709 | 0.722 | 0.601 |
|  | (0.075)+ | (0.672) | (0.675) | (0.675) | (0.725) |
|  |  |  |  |  |  |
| Observations | 4,017 | 4,017 | 4,017 | 4,017 | 3,550 |
| R-squared | 0.027 | 0.037 | 0.043 | 0.045 | 0.057 |
| F test statistics | 4.831 | 4.243 | 3.410 | 3.348 | 2.455 |
| Degrees of freedom | 9 | 16 | 27 | 29 | 46 |
| Robust standard errors in parentheses, *** p<0.001, ** p<0.01, * p<0.05, + p<0.1 | | | | | |
