# Supplementary material for: Health and well-being of male international migrants and non-migrants in Bangladesh: A cross-sectional follow-up study
Source: PLoS Med. 2020 Mar 31;17(3):e1003081. doi: 10.1371/journal.pmed.1003081 (PMC7108692; doi:10.1371/journal.pmed.1003081)
Supplement: S1 Text — (DOCX) [file pmed.1003081.s003.docx]

Proposal submitted to Research and Empirical Analysis of Labor Migration (REALM)

# Project overview

The proposed study will be among the first large-scale representative sample surveys to assess the well-being, living/working conditions and recruitment experiences among a large sample of overseas guest workers. As described in further detail in Section 2, the combined panel dataset from the 1^st^ and 2^nd^ Matlab Health and Socioeconomic Surveys (MHSS2) provides longitudinal data on a representative sample of the population of Matlab, a high out-migration region of Bangladesh with a long history of population research, including full in-person surveys or shorter phone surveys with most overseas guest workers. We propose to enhance these data by following overseas workers excluded from the original sample and by incorporating additional questions on mental health, migrant living/working conditions, and the migration/recruitment process. After outlining our proposed research design, we describe the new data collection and the sample size for the resulting combined dataset. We then provide a timeline for the work.

## 1.1. Research Design

The study has three specific aims, each drawing on a combination of new and existing data. We describe our general research design in the context of Aim 1, before describing Aims 2 and 3 more briefly.

## Aim 1: Measure well-being of guest workers in comparison to non-migrants and internal migrants

Our first and principal aim is to measure the health and well-being of current and former guest workers in comparison to individuals who had not migrated or had migrated internally. Key outcomes of interest include income, mental health, occupational injuries and chronic disease risk factors. We will also examine varitions in mortality, though it is likely that mortality is too rare among guest workers and comparable non-migrants to generate statistically significant results.

Regression analyses will use panel data and account for self-selection into guest worker status using matching procedures and an instrumental variables approach that uses the price of oil and migration-specific social capital as an instrument. This aim will be enhanced by proposed new data collection by increasing the sample size and representativeness, and including more comprehensive mental health measures.

We begin with a simple regression design of the following form

$$Y_{i,2}=\alpha+\beta_{1}*{Mig}_{i,2}+\beta_{2}*I_{i}+\beta_{3}*I_{i,1}+\beta_{4}*I_{i,2}+ \beta_{5}*F_{i,0}+ \varepsilon$$

Where Y is the outcome of interest for person i at time 2 (MHSS2), ${Mig}_{i,2}$ is the respondent’s migration status (overseas internal, internal migrant, non-migrant) at MHSS2, $I_{i}$ are time-invariant individual characteristics (year of birth, religion), $I_{i,1}$ are individual characteristics measured in 1996 in MHSS1, $I_{i,2}$ are individual characteristics measured in MHSS2, and $F_{i,0}$ . are family of origin characteristics measured in 1974.

To better account for self-selection into migration, additional models will take a variety of strategies for accounting for self-selection. First, we will simply assess the possible presence of health selection into migration

$${Mig}_{i,2}=\alpha+\beta_{2}*I_{i}+\beta_{3}*I_{i,1}+\beta_{4}*I_{i,2}+ \beta_{5}*F_{i,0}+ \varepsilon$$

Following the PI’s earlier work using propensity score matching to estimate the effects of migration net of self-selection (Kuhn, Everett and Silvey 2011, Riosmena, Kuhn and Jochem 2016), we will reestimate models using a propensity score matching procedure that accounts for observable sources of self-selection into migration.
