# Supplementary material for: Health and well-being of male international migrants and non-migrants in Bangladesh: A cross-sectional follow-up study
Source: PLoS Med. 2020 Mar 31;17(3):e1003081. doi: 10.1371/journal.pmed.1003081 (PMC7108692; doi:10.1371/journal.pmed.1003081)
Supplement: Attachment — Submitted filename: Response to editor and reviewer comments.docx [file pmed.1003081.s004.docx]

**Requests from the editors:

Please finalize the arrangements for data deposition.**

*MHSS2 data will be deposited with Data Sharing for Demographic Research project (*<https://www.icpsr.umich.edu/icpsrweb/content/DSDR/index.html>) at Interuniversity Consortium for Political and Social Research on February 1, 2020 according to preexisting schedule.

**Regarding data access, you state "No: some restrictions will apply", and we ask you to detail these restrictions.**

Yes. MHSS2 data will available for public download at DSDR, enabling replication of most analyses. The full dataset including MHSS1 and 1982 census baseline controls will be available via restricted access request through ICPSR. This is necessary because earlier versions of these older data are subject to deductive disclosure risks. Therefore the MHSS2 data cannot be linked to them without IRB protection. The only restriction on access to the crosswalk linking MHSS2 to baseline data will be provision of proof of IRB approval from the home institution. Requests will be handled impartially by DSDR and not be the study team, eliminating unnecessary barriers to access.

**We ask you to restructure your title to accord with journal style (non declarative, with a study descriptor). We suggest: "Health and wellbeing of international migrants and non-migrants in Bangladesh: a cross-sectional study".**

Agreed. We are suggesting "Health and wellbeing of international migrants and non-migrants in Bangladesh: a cross-sectional **followup** study". It is a cross-sectional study, but the longitudinal controls allow us to address concerns over self-selection. The inclusion of the word followup draws attention to this.

**Please combine the "methods" and "findings" subsections of your abstract; and add a new final sentence to the new combined subsection summarizing the study's main limitations.**

Done. On limitations, we have written: “Key limitations of this study include the descriptive nature of the analysis, the use of both in-person and phone survey data for international migrants, the long recall period for occupational and mental health risk measures, and the coverage of only one high out-migration sending area.”

**In the abstract, please detail study participants along with summary demographic details. Please also provide quantitative estimates for the main study outcomes, along with 95% CI and p values where appropriate.**

See proposed changes in abstract.

**Please begin the "conclusions" subsection of your abstract with "In this study, we observed that ..." or similar, and condense the subsection.**

See proposed changes in abstract

**After the abstract, we will need to ask you to add a new and accessible "author summary" section in non-identical prose. You may find it helpful to consult one or two recent research papers in PLOS Medicine to get a sense of the preferred style.**

See author summary in text

**We ask you to trim the introduction section of your main text. Around 500 words is usually sufficient; additional material on previous literature can perhaps be relocated to the discussion section.**

Cut to about 560 words.

**Please avoid claims of "the first" or "largest", and where necessary add "to our knowledge" or similar.**

Removed.

**Early in the methods section of your main text, please state whether the study had a protocol or prospective analysis plan, and if so attach the document(s) as a supplementary file (referred to in the text). Please highlight analyses that were not prespecified.**

**We have added this. Analyses were largely prespecified under the funding protocol, though exact dependent variables were not. We have added the following statement to Methods paragraph 3: “**The analysis was carried out with support from the Research & Empirical Analysis of Labor Migration (REALM) program administered by New York University Abu Dhabi. The protocol (included as a Supplementary Appendix) identified the i) statistical methodology, ii) coding scheme for the migration status treatment variable, and iii) control variables, but it did not name the specific dependent variables. Analysis followed the exact guidelines of the protocol, with the exception of restricting the sample to a narrower age range in order to focus on age groups with a non-trivial risk of current overseas migration.”

**Please add a brief statement to the methods section on study ethics approval, whether required or not.**

**Added to Methods paragraph 2.** Protection of human subjects during fieldwork and data analysis were ensured under the icddr,b Ethical Review Committee Protocol #PR-10005.

**Please restructure the early part of the discussion section of your main text so that the first paragraph provides a summary of the paper's findings.**

**Added.**

**Please format reference call-outs as follows: "... in 2018 [1,4].".**

Done. Sorry for that.

**We ask that you revisit the reference list to ensure that all citations meet journal style. Reference 41 may need additional access information to be included.**

Error fixed, along with two others and one stray reference in APA style.

**Please add a completed checklist for the most appropriate reporting guideline, which might be STROBE or RECORD, as a supplementary document (referred to in your methods section). In the checklist, please refer to individual items by section (e.g., "Methods") and paragraph number rather than by page or line numbers, as the latter generally change upon publication.**

**Working on this**

Comments from the reviewers:

***** Reviewer #1:**

**General comment: Why not use some sort of matching (e.g. by propensity score)?  I'm not saying it's required, but was it considered? If so, why was it rejected?**

*We felt that this was an important consideration. We did not present propensity matching results given modest differences in baseline controls and minimal direct associations between baseline controls and many health outcomes, as evidenced by similarity of uncontrolled vs. controlled estimates. The decision was largely to preserve the clarity of the results. But we shared the reviewer’s concern, especially given our past use of matching in migration impact studies. So we did the following:*

1) *Estimated propensity score models using Stata pscore function. We did this for two separate treatments: a) ever overseas migration (meaning both current and returned were taken as treated), b) current overseas migration. We entered all baseline covariates into the first-stage propensity model. Factors such as age, schooling, 1996 assets, parental schooling and father/sibling migration history were highly significant. In each case, the balancing property was satisfied. We observed 98% of all cases in the area of common support.*
2) *We then reestimated all our statistical models dropping cases who were outside the area of common support and then controlling for a factor variable indicating the treatment propensity block based on the first stage to account for propensity. These models yielded changes in coefficient estimates in the range of ±3% of the original effect size, with changes in s.e. only reflective of the slight sample size reduction and no change in significance.*

*We have added the following sentences to the last methods paragraph before dependent variables: We also estimated two-stage propensity score models in which baseline control variables were used to predict selection into international migration. Because this approach did not alter the results, we report only the more intuitive single-stage estimates.*

*We have not added these models to the supplementary appendix, but are happy to do so upon reviewer request.*

**Abstract: It's logistic regression, not logistics**

What an embarrassing typo. We have fixed this.

**There should only be one reference group.**

This is correct. We meant to indicate that we have multiple comparison groups for international migrants. Our models treat international migrants themselves as the reference group in order to facilitate multiple comparisons. We have rephrased this.

**Introduction: "larger representative sample ..."  representative of what?**

Changed to “from a large representative sample of an origin population”


**The formula can probably be deleted. As written, it is incorrect in that some fo the betas are vectors, while the formula implies they are scalars. You would need one beta for each variable, not for groups of variables**

Removed and text adjusted accordingly

**Independent variables (e.g. BMI, grip) should not be categorized. In *Regression Modeling Strategies* Frank Harrell lists 11 problems that this gives rise to and summarizes "nothing could be more disastrous".  I wrote a blog post illustrating some of these, graphically:** [**https://medium.com/@peterflom/what-happens-when-we-categorize-an-independent-variable-in-regression-77d4c5862b6c**](https://medium.com/@peterflom/what-happens-when-we-categorize-an-independent-variable-in-regression-77d4c5862b6c)

I agree. I spend a week of data analysis class covering the perils of categorizing continuous dependent variables, and will use this blog in future classes. We have added mean BMI differences, which are equally striking. We have left the overweight/obese and obese results there since many readers will be more familiar with their meaning and clinical relevance.

For grip strength, we have simply replaced the continuous measure for the <36 gpf cutoff since that cutoff has no particular clinical importance.

**When listing all the IVs, they need to be given operational definitions. That is how were they measured? e.g. what were the choices for "religion"?**

Done.

**Why were fair and poor health lumped together? This is probably a mistake.**

The proportion of respondents reporting poor self-reported health status was just 4%, which seemed low for our models, particularly when using predicted probabilities. We note that fair and poor health are combined in many studies of younger populations for this very reason.

***** Reviewer #2:**

**This study represents important advances in research of migrant health.  There are very few studies that are able to compare migrants, non-migrants and returned migrants from the same community; this study does so using very high quality longitudinal data from a long-standing Demographic Surveillance System. The focus of the study is on migration from South Asia to the Middle East - one of the very established routes for labour migration, but also a very dangerous one for migrants, entailing brutal work conditions, lack of access to services, servitude to an employer, and high risks of death and injury.**

Thanks. We feel very lucky to have had the opportunity to collect and analyze these data.

**1.      The paper highlights higher risks of depression among migrants, but these seem to be non-significant in Table 6.**

The reviewer’s intuition that migrant effects were non-significant was probably driven by a typo in the notes, where we said that stars indicated “statistical test of difference from non-migrant”. This should have said “statistical test of different from current international migrant” and has been corrected. As such, we observe that non-migrants are significantly better off on sadness items and that internal migrants are significantly better off on total depression and sadness.

**2.      With respect to the models, it would be useful to first estimate an interaction model, in which all respondents are pooled and migration status is interacted, for example with sadness items.  This would allow the authors to directly estimate the effect of migrations status.  It would also indicate where stratified analyses are needed.**

Again I think this results from our lack of clarity, unless I misunderstand the concern. All results are based on single regressions that directly estimate the effects of migration status. There are no stratified models. We have clarified the text to make this much clearer.

**3.      When we think about poor health of guest workers, we generally think about injuries and harm directly resulting from the labour migration.  This paper does not find evidence of such problems, but instead finds evidence of overweight and hypertension (though this is also not significant).  These are different types of health risks, and are not directly hazards of the work migrants are doing, but likely rather a bi-product of integration into the host society. It would be informative if the article also indicated the levels of overweight in the host country - do migrants just look like the population into which they are integrating?  If so, this should be discussed as a component of integration foremost, and less so as a hazard of migration.**

This is a truly important question about whether our results reflect exclusion, integration, or segmented integration. Our future research will address this question head-on based on country of destination, duration and level of integration. For now, I don’t think we can provide any simple clarity simply based on destination. Just as one example, BMIs are significantly higher (~24 kg/m2) in Saudi Arabia and UAE, where local populations have high BMIs but migrant exclusion may also be worse. Migrants tend to have lower BMIs of ~23 kg/m2 in Singapore and Malaysia, where local BMIs are lower and migrant regimes may be somewhat more liberal. But BMIs are also relatively lower for migrants in other GCC countries (e.g. Kuwait, Oman) where the host population also has very high BMI and inclusion may be slightly better than in Saudi Arabi and UAE. In other words, any critical tests of weathering vs. assimilation must be reserved for further studies.

For now, we have simply toned down our messaging about depletion/weathering in the discussion and instead said “In the process, their health may be compromised by the physical burdens of their work and by the psychological burdens of separation from family and home society. Migrant health may also be influenced by the complex mix of assimilation into local dietary and health-seeking behavior patterns while also being excluded from key societal and health system functions.”

**4.      The interpretation in the abstract goes further than that in the paper in describing the depletion of migrants, and seems to go beyond the findings of the paper.**

See above.

**5.      It seems there is something interesting going on with internal migrants.  Either in this paper or another, it will be an interesting to examine.**

Agreed. We intend to focus more on internal migration in a paper that uses detailed neighborhood and network data that we have for internal migrants.

***** Reviewer #3:

This study benefits from well described migrant vs non-migrant groups with adequate follow-up over time. That is a unique and major strength of the study. Below some feedback to constructively contribute to improve this work.

1. Methods: "Along with predicted values/probabilities and confidence intervals, we report statistical tests of difference in which current international migrants are the reference category, thereby comparing international migrants to all three counterfactual comparisons groups of non-migrants, internal migrants, and returned migrants." Returned migrants are not described in Table 1. The reason I mention this is because returned migrants can be internal or international returning migrants, so clarification is needed. Later on, it reads "Among respondents in Bangladesh, returned international migrants were identified based on a self-report of whether they had lived abroad in the past five years..." Best to clarify throughout the text.**

In all cases we are referring to returned international migrants. We have updated the text throughout the paper to refer to “returned international migrant” and “current international migrant” where relevant.

**2. Table 1 would be better to be transformed into a Flowchart, to comply with STROBE recommendations, and in that flowchart add how many people were evaluated by what method (in-person or telephone) and how much of the actual data was measured vs self-reported.**

Done, and thanks, this makes the paper much clearer.

**3. Depression. In methods the authors say that "Because there is no validated diagnostic cutoff for depression using this abridged scale, we estimated models for standardized depression scores." What were those scores? How do they compare or related to the existing literature? Are they comparable to other definitions of depression and the cut-offs used? Possibly, given these differences, the authors are better off not labelling this as depression (which has a gold standard definition) but as depressive symptoms. All of this should be clear in methods and also described as a limitation.**

We have changed to depressive symptoms. In describing the limitations of our measures, we have added the following clause “Third, the use of a combination of in-person interviews during festivals and phone surveys created important gaps in the availability of objective health measures for phone respondents, in the need to use a shortened 12-item CES-D module, and in the validity of mental health data for festival respondents.

**4. Hypertension. Why do the authors used 130/80 mm Hg as the cut offs? Explain in the manuscript. Is that cut off applicable to Bangladesh population?**

We follow the 2017 ACC/AHA guidelines for definition of hypertension, which have been employed in a number of studies in Bangladesh. We have added a reference to support this. While no studies have validated the use of this threshold, the general tendency in Bangladesh towards higher CVD risk at even low levels of risk would seem to point to the logic of using 130/80 rather than the previous 140/90.

**5. On mortality. When the authors say, in the Discussion, that "This does not mean that injury risks were low among international migrants, but rather that the risks of injury are extraordinarily high in Matlab and throughout Bangladesh (41)", this reviewer wonders if a similar reasoning could be applied to the mortality indicator. Also, on the mortality, is is something that would require longer follow-up periods to observe differences? What if the accumulated risk factors —higher obesity, hypertension and depression scores among international migrants— would turn into higher mortality later on? Some reflection on this would be ideal.**

This is an important point that we want to get right. There is some similarity between the injury situation and the mortality situation, but I think we want to be clear that the mortality risks were substantially lower for current international migrants, and that this is a contrast to the injury risks, which were broadly comparable to in Bangladesh and quite high by global standards. We have changed the following sentence on the mortality: *“We found that the annual hazard of mortality in the years following international migration was about 75% lower in years spent overseas than in years spent in Bangladesh, and comparable to average annual death probabilities for typical host country populations.”*

The reviewer is also correct that the particular mortality analysis does not reflect long-term consequences. We make this clear in the paragraph on obesity and hypertension risk, where we add: “they nonetheless raise cause for concern that should be addressed in long-term followup studies of health and mortality.”

**MINOR COMMENTS
6. Introduction and discussion. It could benefit from more recent bibliography. See at the end some suggested references for the authors to consider.**

Thanks! We have added several of these

**7. Some clarification of the type of location of internal migration and how much similar were they to Matlab? This is important to understand, a bit, the context of the internal migrants.**

We added “Internal migrants were identified based on area of residence outside the district, with the majority moving for work to major cities such as Dhaka and Chittagong and others moving to nearby towns.”

**8. On the reference group. See comment #1 and contrast it with this sentence from Findings: "Table 3 compares the baseline characteristics of migration status groups. On most characteristics, international and internal migrants were comparable, making internal migrants a relevant reference group." Avoid confusions.**

Changed “international migrants” to “current international migrants” here and in all similar contexts

**9. Typo: mich. "International migrants were mich more likely to have a father who had lived abroad."**

Fixed. Thanks.
